# Computed tomography recent history and future perspectives

**DOI:** 10.1117/1.JMI.8.5.052109

**Published:** 2021-08-11

**Authors:** Jiang Hsieh, Thomas Flohr

**Affiliations:** a GE Healthcare, Waukesha, Wisconsin, United States; b Siemens Healthineers, Erlangen, Germany

**Keywords:** helical and spiral, multi-slice, wide-cone, dual-source, spectral, photon-counting

## Abstract

**Purpose:** We provide a review of the key computed tomography (CT) technologies developed since the late 1980s and offer an overview of one of the future technologies under development. The focus of this review is mainly on the hardware and system development. The topics on the historical event linked to the early days of CT development and other innovations that contributed to the CT development, such as advanced image reconstruction techniques, are covered by companion papers in this special issue.

**Approach:** The review is divided into five major sections, each linked to a key innovation in CT: helical spiral data acquisition, multi-slice CT, wide-cone CT, dual-source CT, and spectral CT. Given the limited scope of this review, only one of the future technologies, photon-counting CT, is discussed in detail. Whenever possible, both theory of operation and clinical examples are provided.

**Results:** Theoretical analyses, phantom results, and clinical examples clearly demonstrate the efficacy and clinical relevancy of five historical technology developments and one future technology in CT. These technologies have improved and will continue to improve CT performance in terms of isotropic volume coverage, improved temporal resolution, and material differentiation and characterization capabilities.

**Conclusions:** Over the past 30 years, technological developments of CT have contributed to the success of CT in many clinical applications such as trauma, oncology, cardiac imaging, and stroke. Advanced clinical applications have and will continue to demand more advanced technology development.

## 1 Introduction

Few predicted the tremendous growth of x-ray computed tomography (CT) technology, and even fewer foresaw the rapid development of clinical applications of CT back in the late 1980s. As a matter of fact, there was so much enthusiasm around the newly introduced magnetic resonance (MR) imaging that predictions of MR taking over CT imaging were accepted by many. More than 30 years later, CT has not only survived the challenges from other imaging modalities but has moved to the frontline of hospital’s diagnostic imaging.

There are many factors that contributed to the success of CT, and there are multiple ways to summarize the technological advancements over the past 30 years. These advances can be examined based on their clinical impact, performance improvements, or the underline technologies themselves. From a clinical impact point of view, coronary CT angiography (CCTA) is no doubt one of the major driving forces for many technological developments. It demands fast data acquisition to freeze the heart’s motion, superior spatial resolving power to characterize small pathologies, and sufficient coverage to enable imaging of the entire heart over one or a few cardiac cycles. Nearly all technological advancements over the years have contributed in one way or another to the success of CCTA today. Of course, stringent requirements for other clinical applications, such as trauma, oncology, and stroke, also played key roles in the technology development.

From a CT performance point of view, technology advancements can be classified chronologically into three major categories: isotropic volume coverage, superior temporal resolution, and spectral information for material classification and differentiation. The initial CT development was focusing mainly on producing good images for a static object: achieving organ-in-a-breath-hold coverage with the introduction of helical/spiral data acquisition and later, isotropic spatial resolution of the entire body with the introduction of multi-slice CT. The next frontier of the development focused mainly on the temporal aspect of the scanning: freezing patient motion and obtaining dynamic information of larger scan ranges with faster gantry rotation, wide-cone CT, dual-source CT (DSCT), larger helical pitch, and advanced algorithms. The third frontier of the CT development was to go behind pure anatomical imaging and provide “color” to the CT images by leveraging the dual-energy or multi-energy data acquisitions. These data acquisition modes are built upon all the previous advances in the category of isotropic volume coverage and temporal resolution improvement.

From the point of view of underline technologies, CT advances can be categorized into five major developments: helical/spiral data acquisition, multi-slice CT, wide-cone CT, DSCT, and spectral CT. Although there are some overlaps among different categories, the separation is somewhat clearer as compared to either the clinical impact perspective or the performance perspective. Therefore, we take this approach to outline the CT development history.

The second objective of this paper is to provide a future perspective on CT. Needless to say, the future technology is both exciting and diverse. Nowadays, artificial intelligence (AI) and deep learning technologies have become, and will continue to be, a powerful tool and a disruptive technology that pushes the frontier of CT. AI-supported improvement and automation of the CT scan workflow and approaches to enhance the clinical information of CT images have changed the way technicians and radiologists work. Modern CT scanners provide anatomy-aligned reconstructions and advanced visualizations as part of standard image reconstruction tasks, and even automated identification and quantification of pathological processes are on their way to routine integration into the CT workflow. On the information presentation front, additive manufacturing [or three-dimensional (3D) printing] will continue to impact the way radiologists interact with other medical professions and patients. The rapid development of virtual reality and augmented reality has and will continue to impact many radiology departments, ranging from training to operation, and new workflows. Interestingly, despite all technological advances and improvements, all modern CT scanners are still based on the third-generation rotate–rotate geometry. There are new developments on the x-ray tube technologies that may allow multiple x-ray sources to be placed on the same CT gantry and potentially lead to a new generation of CT scanners with less or no mechanical motion. Other approaches, such as phase contrast CT, have recently gained considerable attention in the scientific literature but have not yet led to clinically useable whole-body CT systems. The list can go on and on. Given the limited scope of this reviewing article, it is impossible to cover all aspects that enabled the growth of CT. Therefore, the last section is dedicated to only one of the upcoming CT technologies: photon-counting CT.

Finally, this paper focuses mainly on the development of CT technology since the late 1980s. For historical events linked to the early days of CT development, a companion paper in the same issue provides an excellent review.^1^ In addition, this paper focuses primarily on the major hardware or system developments in CT. Other innovations that contributed to the CT development, such as advanced image reconstruction techniques, are covered by another companion paper and will be omitted here.^2^

## 2 Spiral or Helical CT

In 1990, CT scanners with continuous gantry rotation enabled by slip-ring technology and continuous patient transport during data acquisition were introduced by major vendors. This so-called spiral or helical data acquisition^3^^,^^4^ constituted a fundamental evolutionary step in the development and ongoing refinement of CT imaging techniques. For the first time, volume data became available: whole organs could be covered in a single-breath-hold without misregistration of anatomical details and overlapping images could be reconstructed at arbitrary z positions. This was a major achievement compared to previous step-and-shoot data acquisition techniques which provided only a few slices for the organ of interest. Volume data became the very basis for applications such as CT angiography, which has revolutionized assessment of vascular disease. The ability to acquire volume data also paved the way for the use of 3D image processing techniques such as multi-planar reformations, maximum intensity projections, surface shaded displays or volume rendering techniques (VRT) in CT.

**Fig. 1:**
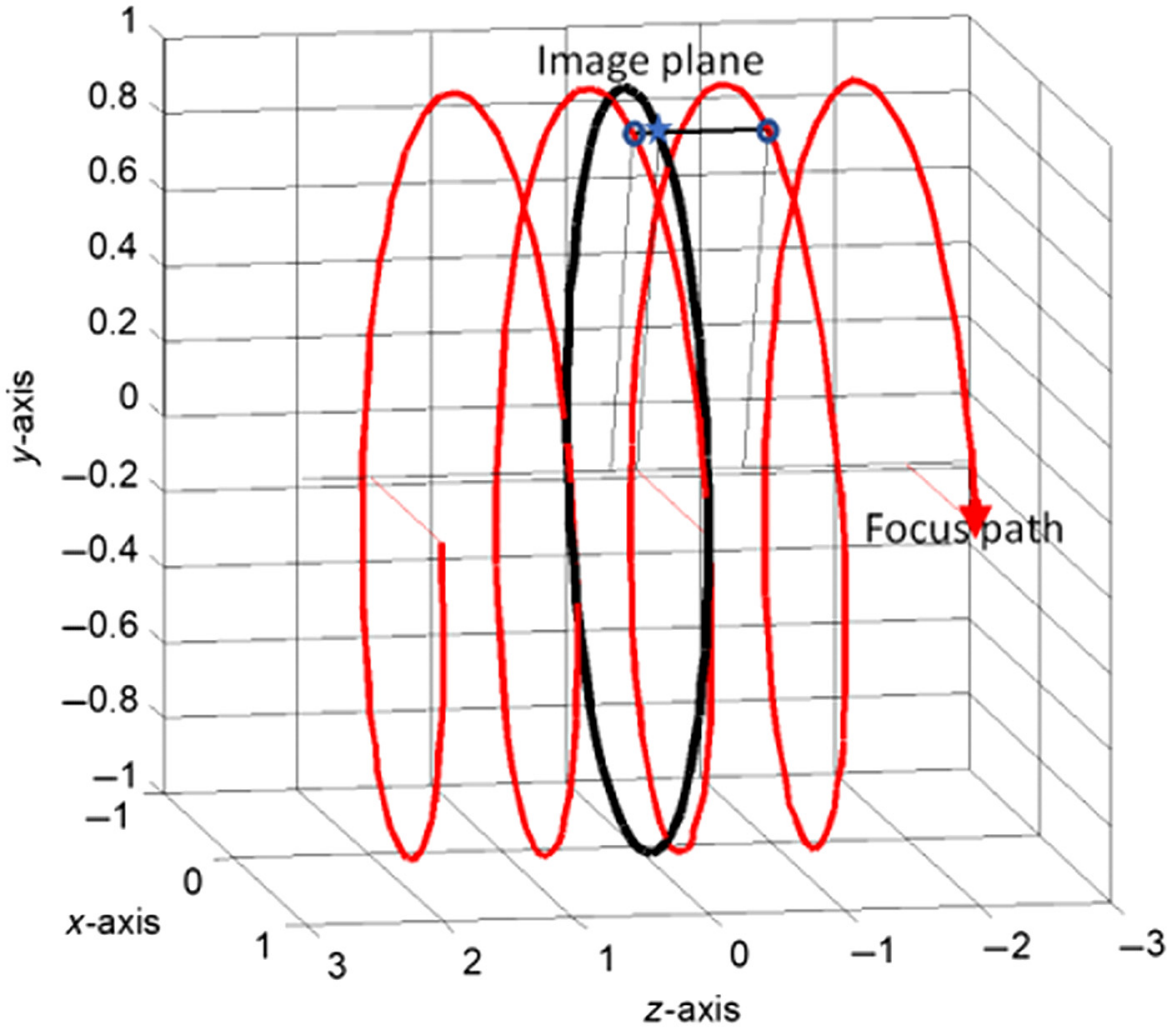
Spiral interpolation to the plane of the black circle with algorithm 360-LI.

When it was introduced, spiral CT made new demands on image reconstruction. The scan geometry in spiral CT is non-planar. The tube does not move on a circular path, it rather moves along a helical path. In spiral CT, it is convenient to characterize the motion of the patient bed by a dimensionless figure. The so-called pitch p is defined as the ratio of table feed per rotation and collimated beamwidth. In Fig. 1, the helical path of the focal spot is displayed in a 3D view. The black circle represents all focal spot positions needed for image reconstruction in the corresponding plane. Unfortunately, measured data in this plane are available only for one projection angle: the intersection point of the helical path with the black circle. For image reconstruction, we need to provide data for the other projection angles as well for position *. For this angle, measured data are available at z positions in front of and behind the plane of interest. Data at position * can be obtained by a linear interpolation along the line connecting the points marked by o, *, and o in Fig. 1 (360-LI interpolation).

The 360-LI algorithm is computationally simple, yet it has some drawbacks. Since the interpolation inputs (partners) of this 360-LI algorithm are 360 deg apart, data are needed from 360 deg in front of the reference plane to 360 deg behind the reference plane. In total, this comprises an angular data range of 720 deg. This introduces a challenge in higher pitch because of the large z distance between the interpolation inputs (partners). To reduce this distance, interpolation inputs (partners) closer to the reference plane need to be identified. Indeed, for each ray, an interpolation partner is already available after approximately half a rotation, when x-ray tube and detector have interchanged their positions. This is the so-called “complementary ray,” and the spiral interpolation scheme relying on direct and complementary rays is called 180-LI.^5^

**Fig. 2:**
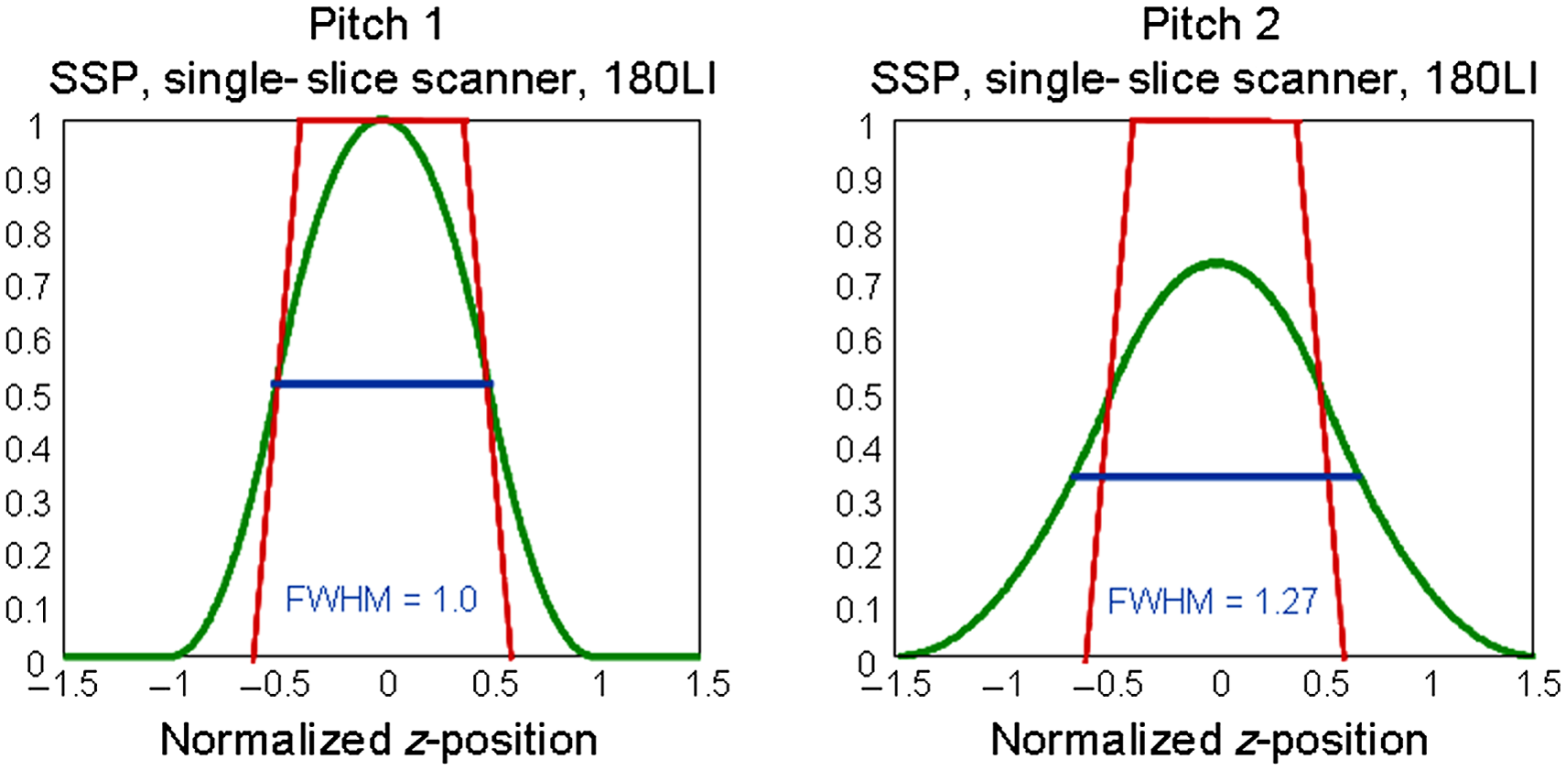
Effective slice-width in spiral/helical CT: the collimated slice profile, indicated in red, is a trapezoid. The slice profiles after spiral/helical interpolation are bell-shaped (see the green curves for the most commonly used 180-LI approach at different pitch values).

As a consequence of spiral interpolation, the slice profile changes from the trapezoidal, in some cases from an almost rectangular shape known from axial step-and-shoot scanning to a more bell-shaped curve, as shown in Fig. 2. The 180-LI algorithm leads to narrower, better defined slice profiles than 360-LI at the expense of increased image noise.^5^ The image noise with 360-LI is lower than with step-and-shoot data acquisition; with 180-LI, it is higher.

**Fig. 3:**
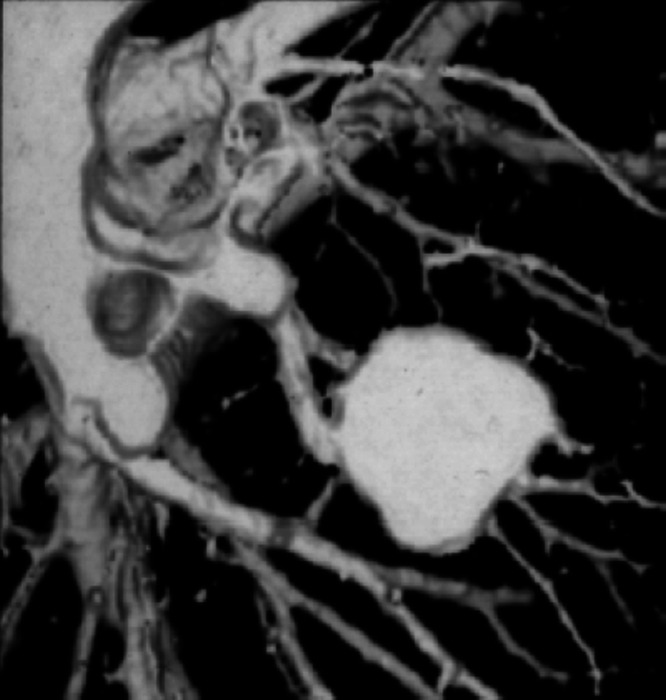
First spiral CT scan of the lung presented at RSNA 1989 (courtesy of Willi Kalender).

The z axis resolution is no longer determined only by the collimated beamwidth (as in axial step-and-shoot scanning) but by the effective slice width $s_{\mathrm{eff}}$ as a result of spiral interpolation. Usually, $s_{\mathrm{eff}}$ is defined as the full-width-at-half-maximum (FWHM) of the slice sensitivity profile. For both 360-LI and 180-LI, $s_{\mathrm{eff}}$ increases with increasing pitch p, albeit much less for 180-LI. This is a consequence of the increasing longitudinal distance of the projections used for spiral interpolation. Figure 3 shows an early example of a clinical spiral CT scan.

## 3 Multi-Slice CT

**Fig. 4:**
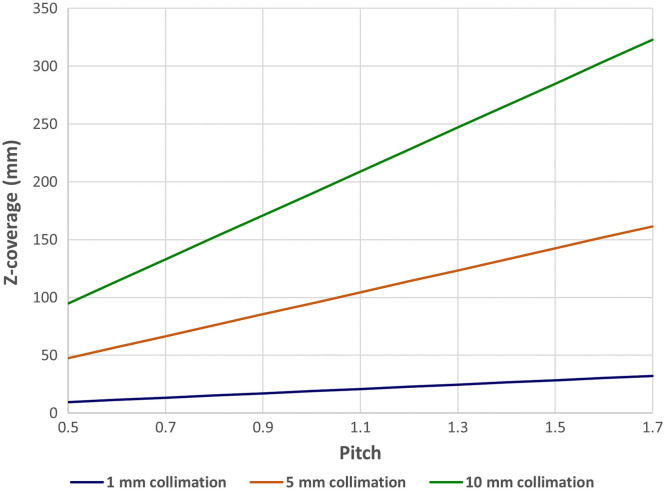
Z coverage as a function of helical pitch with a 20-s breath-hold at 1-s gantry rotation speed.

The introduction of helical/spiral CT enabled the organ coverage in a single-breath-hold. However, when the size of the organ along the patient longitudinal axis (z axis) is large, we are forced to make a trade-off between the amount of coverage in z and the spatial resolution (slice thickness). As the desired volume coverage increases, the slice thickness of the reconstructed images increases almost linearly. For illustration, Fig. 4 depicts the z coverage as a function of helical pitch for a CT system with gantry rotation speed of 1.0 s and a patient breath-hold time of 20 s. For small organs, such as the inner auditory canal that spans roughly 30 mm in z, a slice thickness of 1 mm can be achieved with a helical pitch of 1.5 (black curve). For a complete coverage of the lung with a typical span of 300 mm in z, however, the image slice thickness needs to be increased to 10 mm (green curve), whereas a helical pitch of >1.5 needs to be used.

**Fig. 5:**
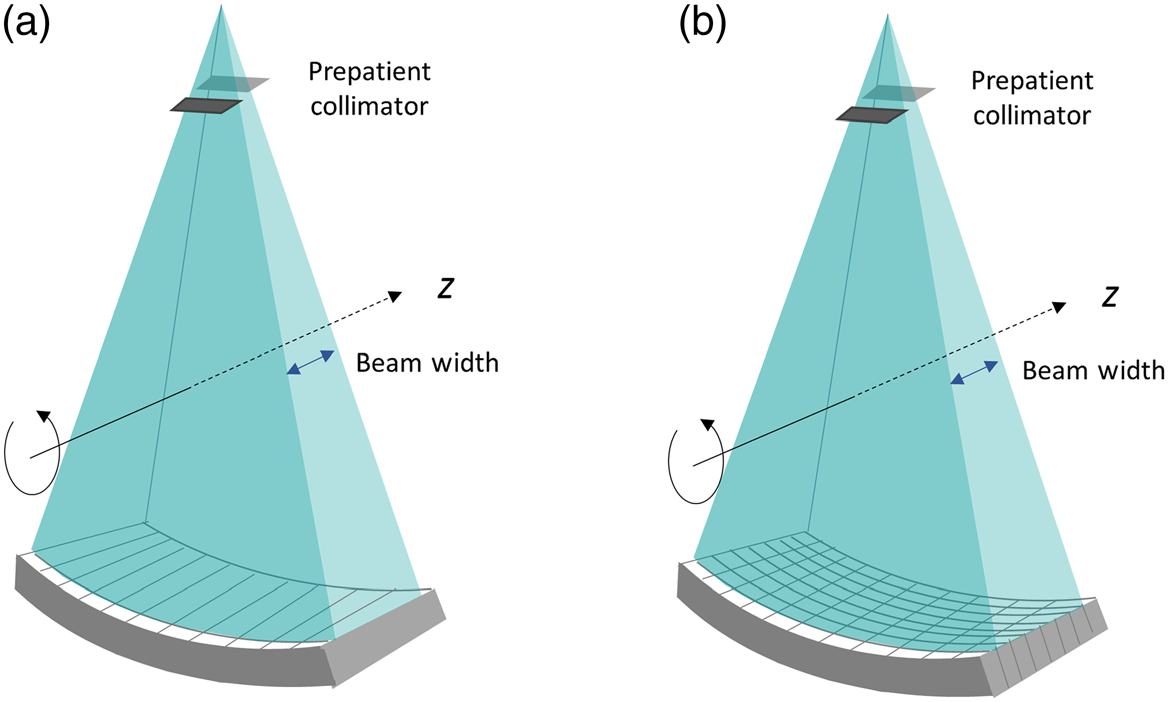
Illustration of different CT geometries: (a) a single-slice CT and (b) an eight-slice CT. For the single-slice configuration, the slice thickness is defined by the prepatient collimation (beamwidth) while for multi-slice system, the slice thickness is defined by the detector row width.

Such a trade-off stems from the fact that the slice thickness of a single-slice CT system is mainly determined by the width of the prepatient collimation (therefore the width of the x-ray beam), as shown in Fig. 5(a), since there is no capability of the detector to differentiate the location of the x-ray interaction along z. All x-ray photons registered in a detector cell will be summed together regardless of where they land. When larger volume needs to be covered for each projection, the slice thickness naturally suffers. Such constraint can be reduced or eliminated by modifying the detector design such that the slice thickness of the image is independent of the primary x-ray beamwidth, as shown in Fig. 5(b). This figure illustrates an eight-slice CT detector where each original single-slice detector cell is divided into eight pieces with each element being read out independently. Consequently, the z location of the x-ray photons is identified by individual detector cells such that the slice thickness of the image becomes independent of the x-ray beamwidth.

Although several earlier CT scanners had two detector rows, starting with the first CT scanner in 1971,^1^ and followed by a two-row scanner introduced in 1992 (Elscint CT Twin), it is generally agreed that the multi-slice scanners that significantly impacted the clinical practice were introduced in 1998 when the four-row detector systems became clinically available. Since then, the number of detector rows increased quickly from 4 to 8, 16, 32, and 64. With an increase in the number of detector rows, the width of the detector row (in z) also reduced from over 1 mm to sub-mm. It is worth pointing out that in the early days of multi-row detector design, the detector row width was not necessarily uniform along the z direction. For example, a 16-row detector may contain 16 sub-mm detector rows at the center and 8 detector rows with doubling the width on both sides. When sub-mm imaging is desired, the prepatient collimator limits the x-ray beamwidth in order to irradiate only the center 16 sub-mm rows. When wider coverage is desired, the prepatient collimator opens to illuminate all detector rows, and sub-mm rows in the center are summed pairwise to match the outer detector row width. Such design was not necessarily by choice, it was mainly driven by limitations on electronic packaging and bandwidth of the data transmission. With the introduction of 64-row detectors, however, the mix-mode detector design was pretty much eliminated.

**Fig. 6:**
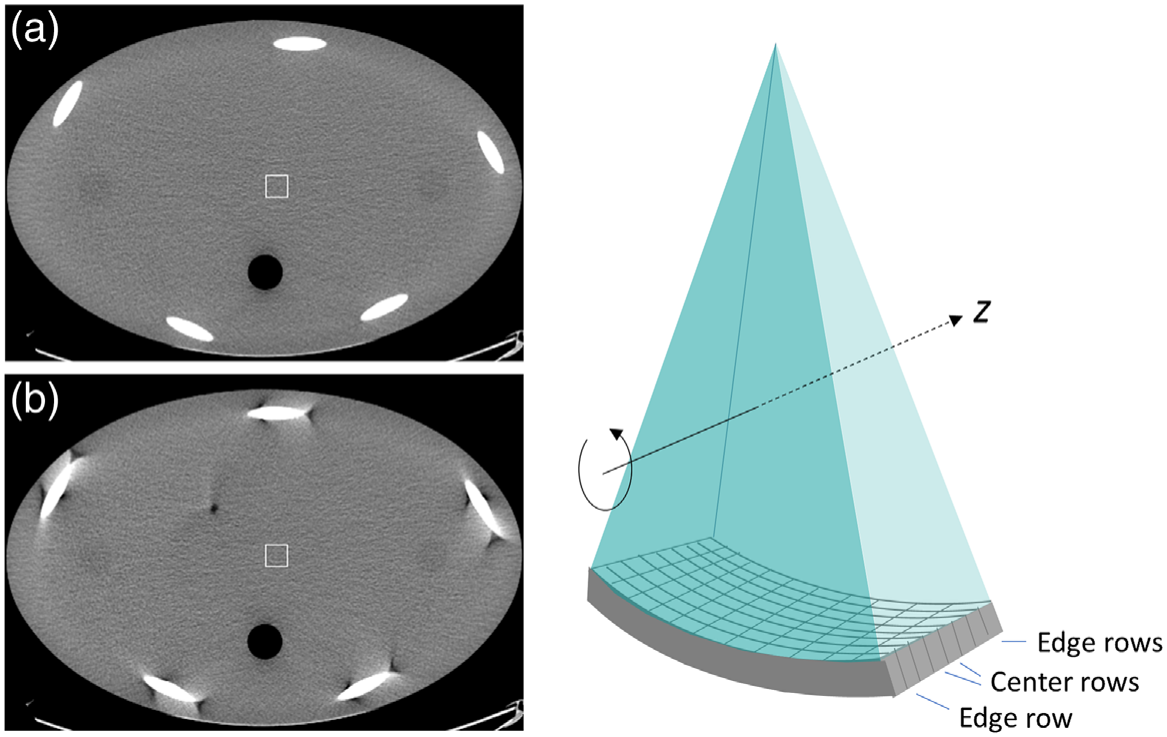
Illustration of cone-beam artifact with a helical body phantom. Images were acquired in a step-and-shoot mode data acquisition. (a) The center row location and image and (b) an edge row position.

For MDCT, reconstruction algorithms are more complex as compared to the single-slice CT.^6^^–^^10^ Research activities were focused on reducing the cone-beam-related artifacts for step-and-shoot mode data acquisition and “exact” reconstruction algorithms for helical type of data acquisition. An example that illustrates the cone-beam artifact is depicted in Fig. 6. For the image acquired with center detector rows in a step-and-shoot data acquisition [Fig. 6(a)], x-ray photons detected by these rows can be approximately assumed to be traveling parallel to the x−y plane, while for the image acquired at the edge rows x-ray photons travel at oblique angles relative to the x−y plane and such an approximation is no longer valid. The cone-beam effect increases as the detector coverage increases in z. Consequently, reconstruction algorithms have expanded from 2D to 3D and additional compensation is needed to account for the missing information in the data collection. Since there is a dedicated paper on this subject for the special section, detailed analysis and description will be omitted here.^2^

**Fig. 7:**
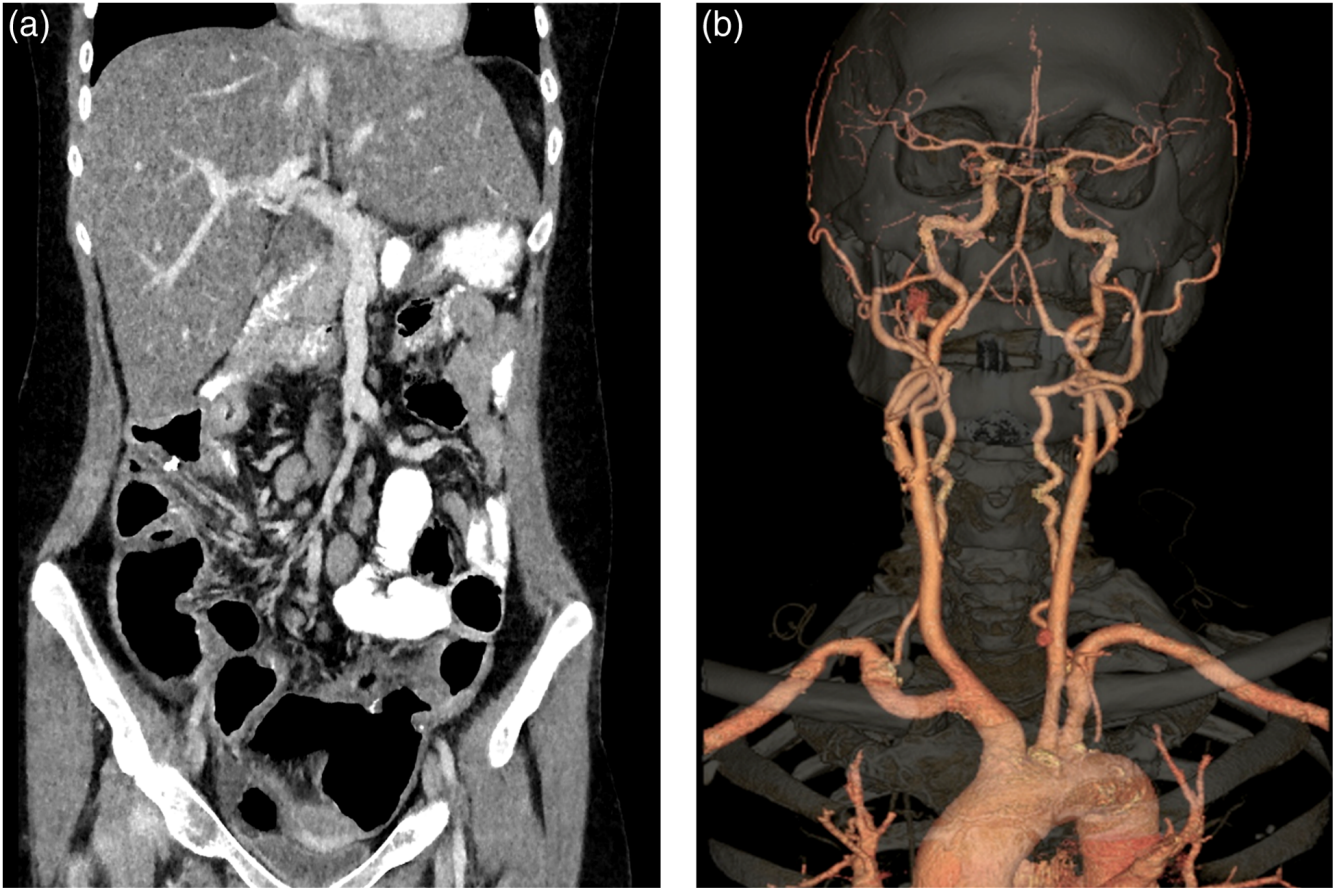
Clinical images acquired on a 64-slice scanner (a) coronal image of an abdomen and pelvis study and (b) volume rendered image of a carotid with circle of Willis CTA study.

With the introduction of multi-slice CT, the goal of obtaining isotropic spatial resolution in x-, y-, and z-directions has been gradually fulfilled. Historically, the in-plane spatial resolution (x−y) was much better compared to the z resolution. Such discrepancy was not an issue in the early days of CT since images were reviewed primarily in the axial orientation. With the introduction of multi-slice, especially with the number of detector rows at or above 16, images are viewed in a 3D manner. For example, sagittal (y−z plane), coronal (x−z plane), and volume rendered images have gained significant popularity and are viewed routinely by radiologists, as shown in Fig. 7.

## 4 Wide Cone CT

**Fig. 8:**
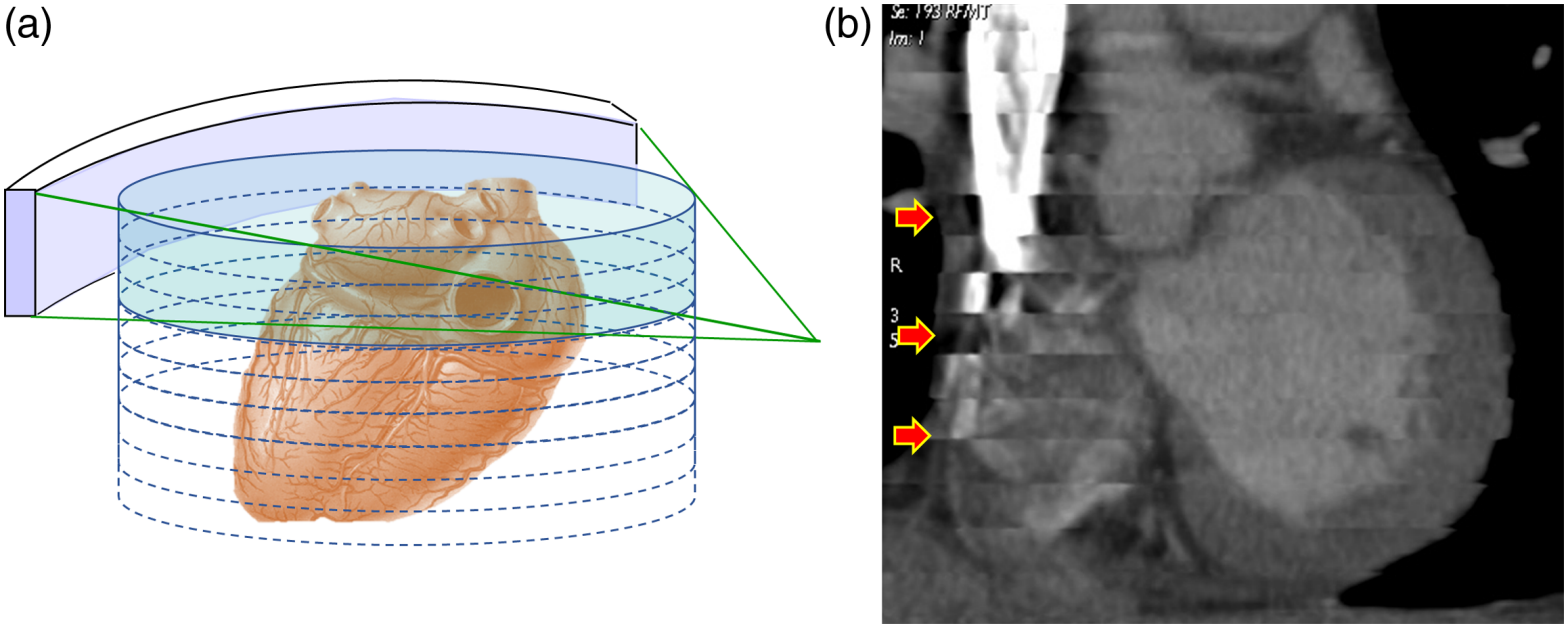
Impact of a narrow detector on CCTA imaging. (a) The detector covers only a fraction of the heart and multiple acquisitions are needed to enable a full coverage. (b) An example of CCTA acquired on a 16-row detector with phase misregistration (red arrows).

The introduction of multi-slice CT has enabled the organ coverage with an isotropic spatial resolution. For temporally sensitive clinical applications, such as CCTA, the ability to capture the entire organ in a single rotation is important. Although a 64-row scanner can acquire good CCTA images, the quality of the obtained image is highly dependent on the patient heart rate and stability. According to *Gray’s Anatomy*, the average heart length is 12 cm.^11^ To cover the entire heart, multiple rotations and therefore multiple cardiac cycles are required. For moderate heart rate, a step-and-shoot mode of data acquisition was proposed, in which a series of slightly overlapped axial data acquisitions is performed over multiple cardiac cycles to achieve low-dose cardiac imaging.^12^ When the heart rate is high and highly variable, a technique called multi-sector reconstruction was often used. In such cases, a small helical pitch (typically around 0.2) is used so that images can be reconstructed with data collected over more than one cardiac cycle with each cycle contributing a fraction of the data required for reconstruction. Since the data collected in each heart cycle is a fraction of the overall required half-scan, improved temporal resolution can be achieved at an increased radiation dose. Due to various hardware limitations, however, neither single-sector nor multi-sector approach is perfect. Clinical examples of suboptimal image quality can be found in either approach. Figure 8(b) depicts an example of banding artifacts caused by inconsistent heart motion over multiple cardiac cycles.

**Fig. 9:**
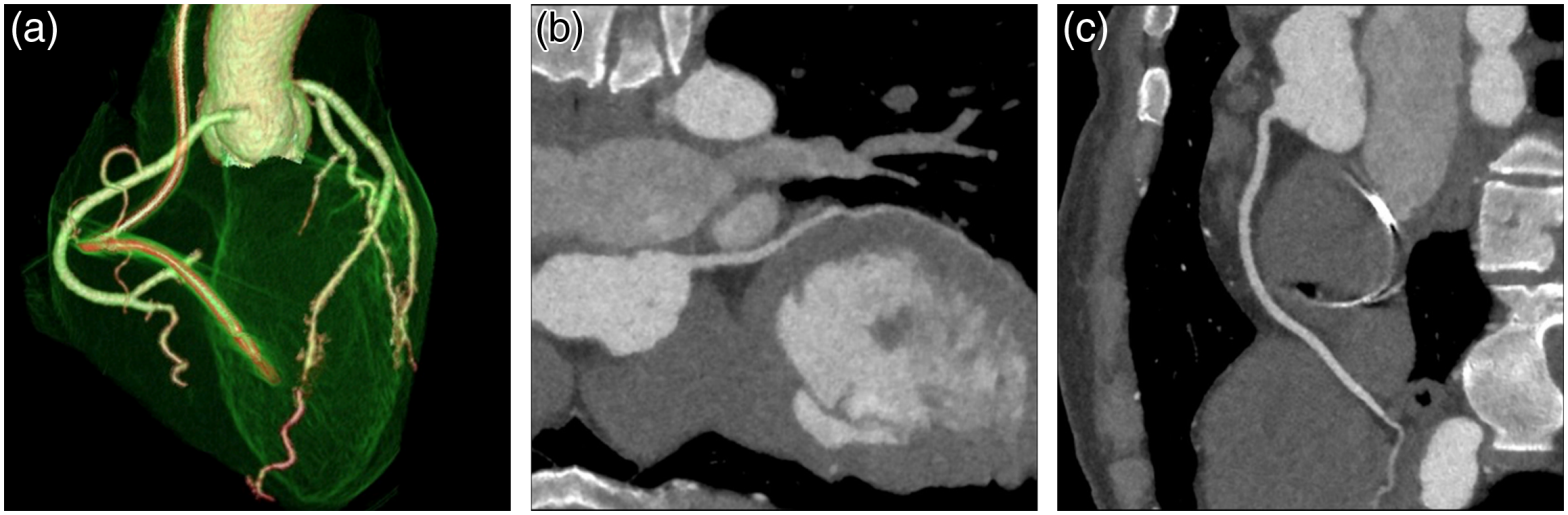
CCTA images of an arrhythmia patient with heart rate varying from 38 to 111 bpm acquired on a 16-cm detector system in one cardiac cycle at 0.6 mSv. (a) 3D volume rendered image; (b) reformatted image of the left anterior descending artery (LAD); and (c) reformatted image of the right coronary artery (RCA). Image courtesy of Prof. Kaufmann, USZ, Zürich, Switzerland.

If the detector coverage in z is large enough to cover the entire heart, all imaging data will be collected within a single cardiac cycle. Under such an arrangement, there is no inconsistency in cardiac phase since the entire heart is acquired in a single heart cycle. Figure 9 depicts a patient CCTA examination with significant heart rate variation (38 to 111 bpm during the examination). In addition, arrhythmia was detected during the initial data acquisition. Since the patient table remains stationary during the entire data acquisition, x-ray exposure was suspended until a regular heart rate was detected. Excellent image quality is achieved despite the patient heart rate instability.

**Fig. 10:**
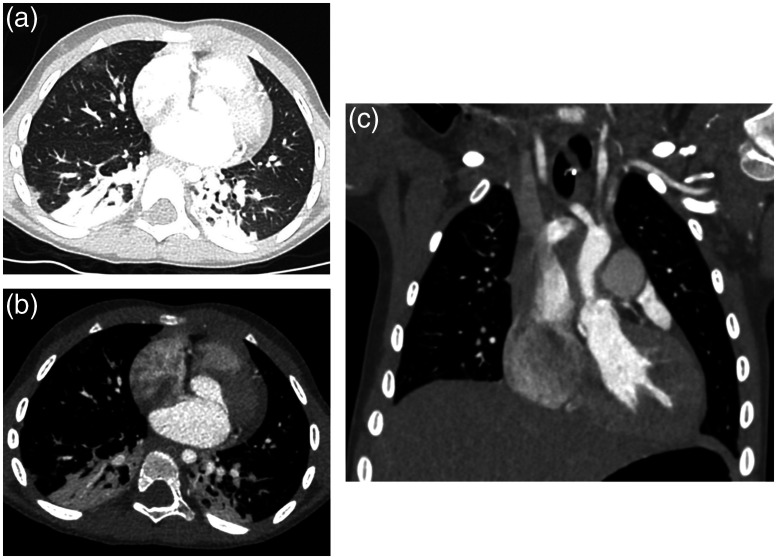
Example of a 3-year-old pediatric patient imaged without sedation (heart rate 117 bpm). A 16-cm gated axial data acquisition at 70 kVp was used (${\mathrm{CTDI}}_{\mathrm{vol}}=1.3\;\mathrm{mGy}$). (a) Axial image displayed with a lung window; (b) axial image displayed with a soft-tissue window; and (c) coronal image displayed at a soft-tissue window. Image courtesy of Dr. W. Dennis Foley, Froedtert and Medical College of Wisconsin, USA.

Of course, CCTA imaging is only one of the clinical examples that benefits from the wide z coverage detector. Other clinical benefits, such as volume perfusion and dose reduction, have been shown by investigators.^13^^–^^15^ At the same helical pitch and gantry rotation speed, a larger coverage detector allows a faster z coverage, which can minimize patient motion-related issues and relax the patient breath-hold requirement for adults and sedation for pediatric patients. Figure 10 depicts a study of a 3-year-old pediatric patient without sedation. Despite the high heart rate (117 bpm), excellent image quality was obtained. The study was carried out using a 16-cm detector system in a gated axial data acquisition. Given the small patient size, 70 kVp was used to optimize the dose performance at 1.3 mGy ${\mathrm{CTDI}}_{\mathrm{vol}}$.

**Fig. 11:**
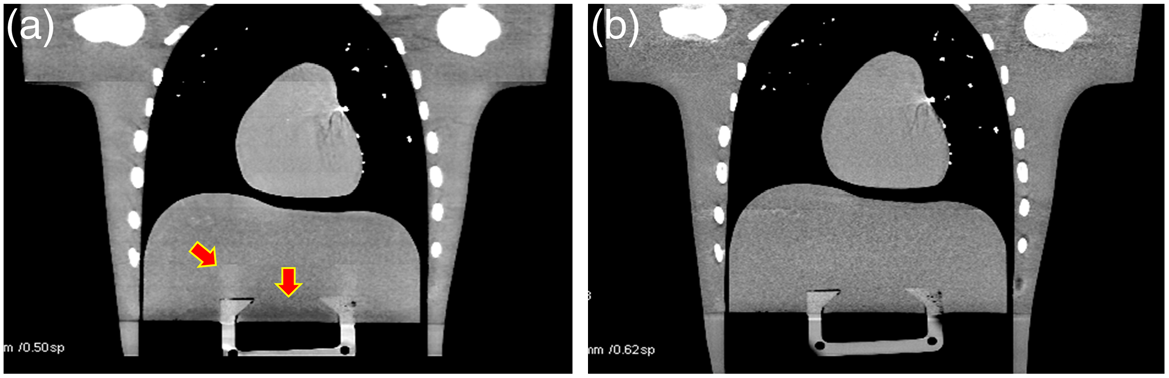
Illustration of the impact of scattered radiation and a hardware approach for scatter rejection: (a) image acquired on a 1D postpatient collimation system with red arrows indicating scatter artifacts and (b) image acquired on a 2D focusing collimation system with additional scatter rejection in z.

There are technical challenges, however, associated with the wide cone CT system.^16^ One of the challenges is the scattered radiation. It is well known that the amount of scatter, more precisely the scatter to primary ratio, increases almost linearly with the coverage in z. Therefore, proper compensation is needed for wide cone CT systems. The solution can come from either the hardware design or algorithmic corrections. For example, since scattered x-ray photons travel in all directions, rejection of scattered radiation should not be limited to the x−y plane. By utilizing 2D focusing postpatient collimation, the scattered radiation along the z direction can be effectively reduced or eliminated. Figure 11 illustrates the impact of scatter along the z axis. When a 1D collimator is used (rejecting primarily scattered radiation in x−y plane), shading and ghost image due to scattered radiation along the z direction can be observed [Fig. 11(a)]. Either prevention or correction approaches can be used for scatter handling. For example, by employing a 2D focusing collimation, a significant portion of the scattered radiation is prevented from reaching the CT detector and image artifacts can be significantly suppressed [Fig. 11(b)]. On the other hand, many algorithmic approaches were developed to compensate for the image artifacts induced by the scatter.^17^^–^^19^

**Fig. 12:**
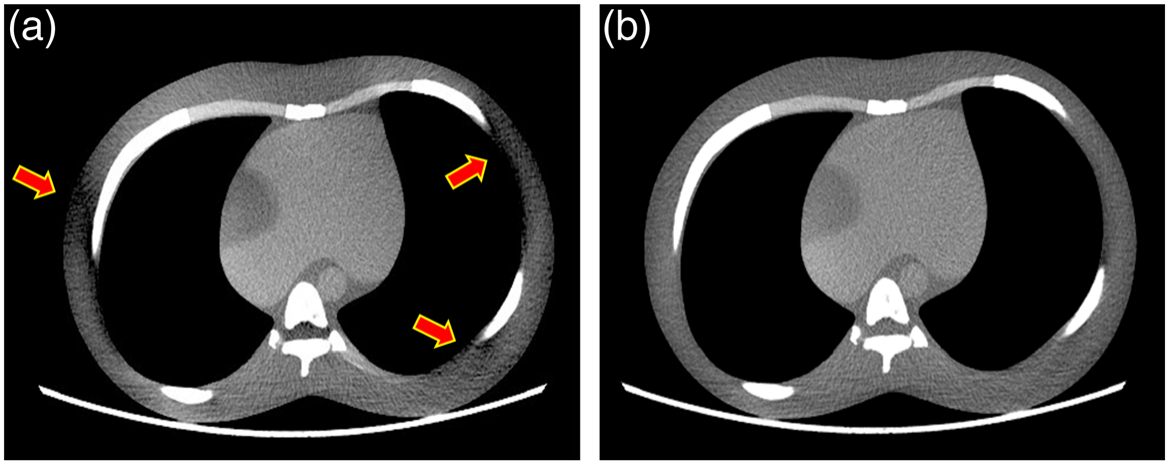
Reconstructed images of a computer simulation of a chest phantom: (a) illustration of cone beam artifact (red arrows) with FDK reconstruction algorithm and (b) artifact suppression with a more advanced reconstruction algorithm.

Another technical challenge is the handling of artifacts associated with the large cone angle. For the step-and-shoot mode of data acquisition, the amount of missing frequency-domain information at the off-centered image location increases with the distance from the center plane, and the sampled frequencies shape like a doughnut. In addition, proper handling of the redundant information in the case of half-scan data acquisition is equally important. In the case of a fan-beam data acquisition (single-slice), a weighting function is applied to ensure the contribution from redundant samples sum up to unity. The compensation for wide cone system needs to be more sophisticated to ensure redundant frequency information is properly compensated. If proper care is not taken, image artifacts can be observed as illustrated in Fig. 12(a) where a simple FDK reconstruction algorithm is used to reconstruct a computer simulated phantom. Extensive research has been conducted on the wide-cone reconstruction algorithms and the topic is well covered by a companion paper in this issue.^2^ An advanced algorithm to compensate for both missing frequency and redundant sample enables a significant reduction or elimination of the artifacts as illustrated in Fig. 12(b).

## 5 Dual-Source CT

**Fig. 13:**
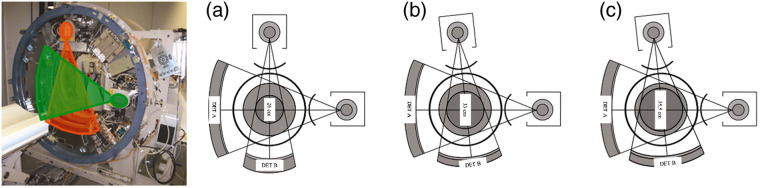
DSCT with two independent measurement systems: (a) first generation with a system angle of 90 deg and (b) second generation. To increase the SFOV of detector B to 33 cm, a larger system angle of 95 deg was chosen. With the third-generation DSCT (c), the SFOV of detector B was further increased to 35.5 cm.

For robust CCTA at higher and irregular heart rates, good temporal resolution is important as well. Temporal resolution in CT is the time needed to acquire enough data to reconstruct an axial image—this is approximately half the gantry rotation time for image pixels close to the isocenter, where the heart is usually positioned (a so-called half-scan). Temporal resolution should not be confused with the total scan time of a CT scan. CT systems have become faster and faster during the past 30 years. Yet, gantry rotation times shorter than 0.25 s, resulting in 125 ms temporal resolution, could not be achieved because of mechanical challenges caused by the enormously increasing centrifugal forces. An alternative scanner concept that provides enhanced temporal resolution but does not require faster gantry rotation is a DSCT; this is a CT system with two x-ray tubes and the corresponding detectors at an angle of about 90 deg.^20^ Both measurement systems acquire CT scan data simultaneously at the same anatomical level of the patient. Since 2006, three generations of DSCT systems have been commercially introduced (see Fig. 13).

**Fig. 14:**
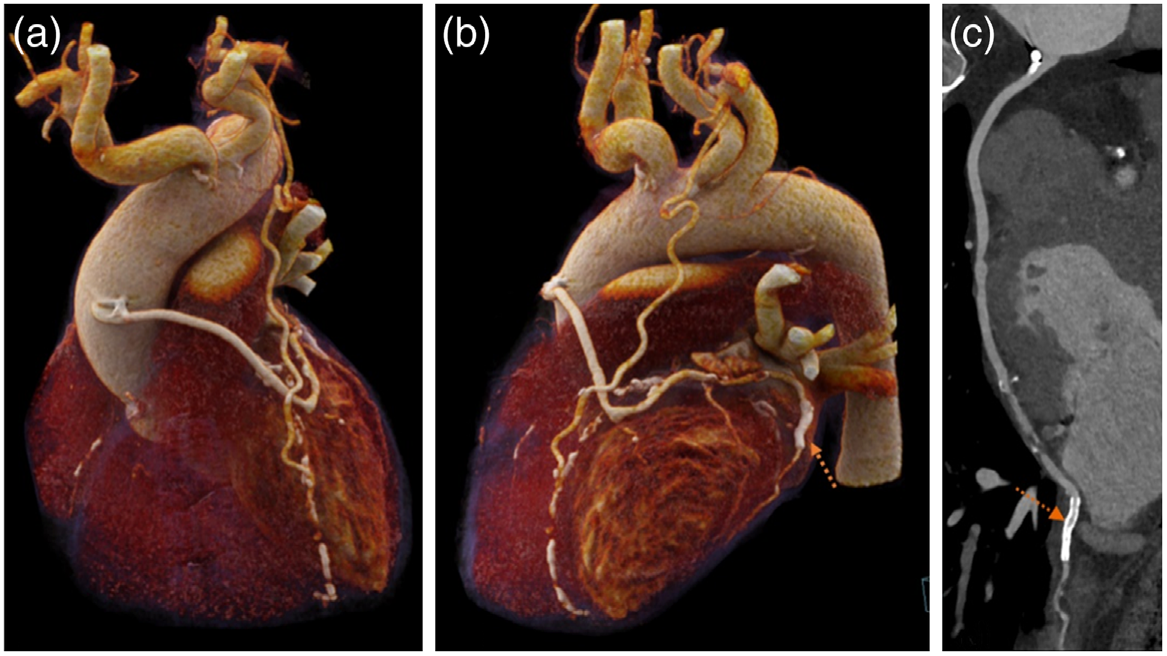
CCTA images of a patient with bypasses and atrial fibrillation with unstable heart rate, acquired on a third-generation DSCT. ECG-triggered sequential scan with a scan range of 21.9 cm. (a), (b) 3D volume rendered images (VRT) and (c) curved MPR. The arrow illustrates a stent. Image courtesy of Peking Union Medical College (PUMC), Beijing, China.

The temporal resolution achieved with DSCT is approximately a quarter of the gantry rotation time. Each measurement system acquires only about 90 deg of scan data, and both data segments are appended to the half-scan needed for image reconstruction close to the isocenter. DSCT provides images with a temporal resolution well below 100 ms—the third-generation DSCT with 0.25-s gantry rotation time as an example achieves 66-ms temporal resolution. Temporal resolution is independent of the patient’s heart rate. This is a major difference to single-source CT systems, which can provide similar temporal resolution by combining data from several heart cycles into one image in a multi-sector reconstruction. With a multi-sector reconstruction temporal resolution strongly depends on the relation of heart rate and gantry rotation time—there are “sweet spots,” but there are also heart rates where no better temporal resolution than half the gantry rotation time can be achieved. Meanwhile, the potential of DSCT to reliably perform CCTA in patients with high and irregular heart rates has been demonstrated,^21^ and it has been shown that DSCT is sufficiently accurate to diagnose clinically significant coronary artery disease in some or all difficult-to-image patients.^22^ Figure 14 shows the clinical example of a coronary CTA examination with a third-generation DSCT, requiring a relatively long-scan range of 22 cm because the patient had coronary bypasses.

**Fig. 15:**
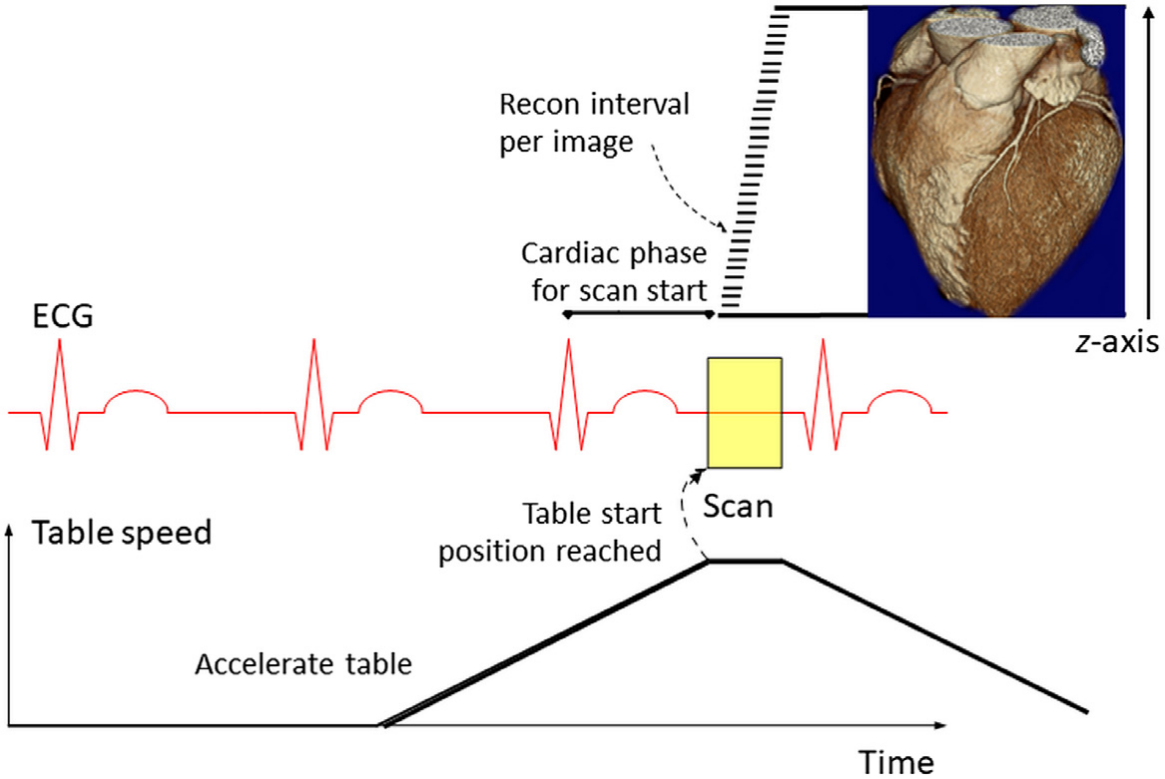
Principle of the ECG-triggered high-pitch scanning with DSCT. The scan data for images at adjacent z positions are acquired within the same cardiac cycle at slightly different cardiac phases. Figure with modifications from Ref. 23.

DSCT systems offer an alternative way to scan the entire heart within one heartbeat. With a single-source CT, the spiral pitch p (i.e., the table travel distance per rotation divided by the detector width at the isocenter) cannot be higher than 1.5 to ensure gapless volume coverage along the z axis. Otherwise, sampling gaps will lead to excessive image artifacts. With DSCT, however, data acquired with the second tube-detector system fill these gaps up to a pitch of 3.2 (again referred to the width of one detector).^23^ This corresponds to a scan speed of 737  mm/s with the third-generation DSCT (57.6-mm detector coverage and 0.25-s gantry rotation time). The high-pitch scan mode can be combined with ECG-triggering (see Fig. 15). Then the entire heart (12 cm) is covered in about 160 ms with a temporal resolution of 66 ms per image. The scan data for images at adjacent z positions are acquired within the same cardiac cycle at slightly different cardiac phases. Successful use of the high-pitch scanning technique for CCTA in patients with sufficiently low and stable heart rates has been demonstrated in several studies, with the potential to scan the entire heart in one beat at a low radiation dose.^24^^,^^25^

**Fig. 16:**
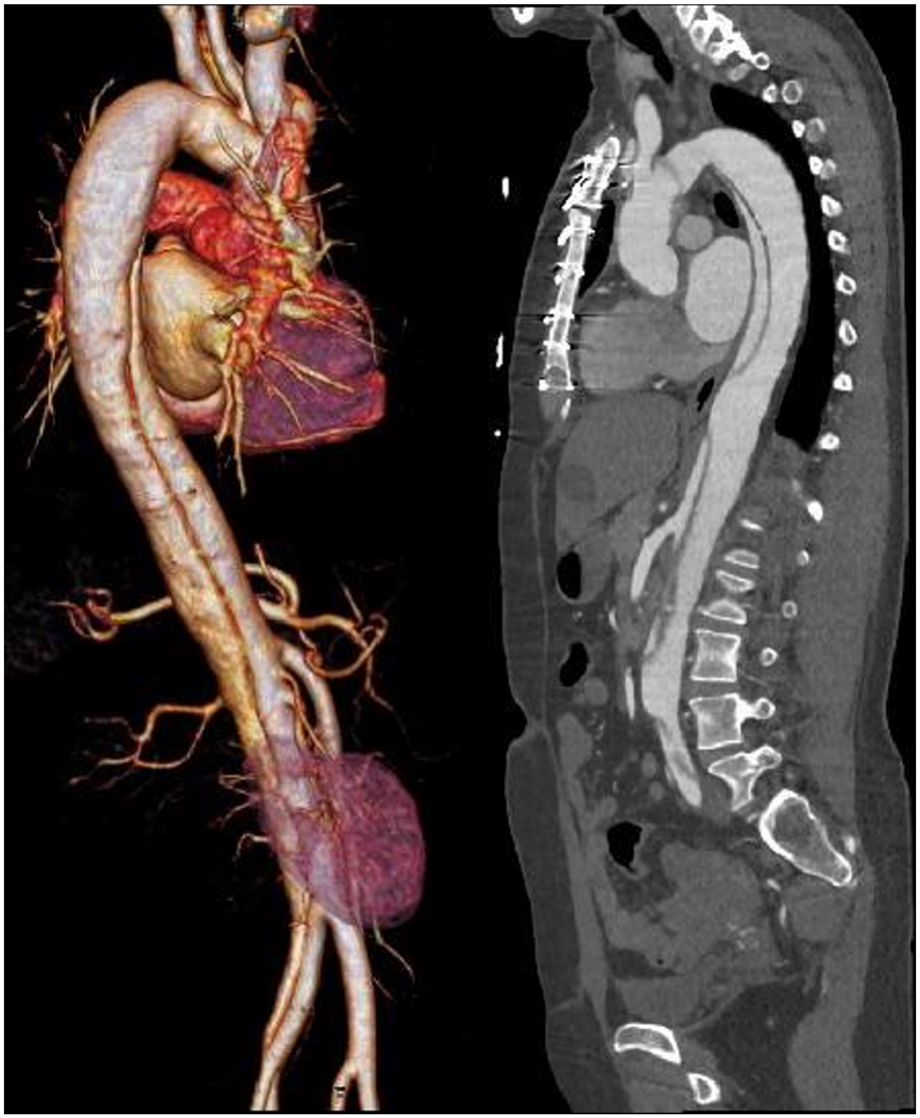
ECG-triggered high-pitch spiral scan of the aorta in an emergency situation: aortic dissection with affected left renal artery; acquired with a third-generation DSCT at pitch 3.2, total scan time 0.8 s, 90 kVp, ${\mathrm{CTDI}}_{\mathrm{vol}}=2.81\;\mathrm{mGy}$, DLP=178  mGycm. Images courtesy of Klinikum Großhadern, Ludwigs-Maximilians University Munich (LMU), Germany.

The high-pitch scan mode is beneficial for the examination of larger anatomical ranges in very short-scan times, such as chest CTA at high temporal resolution and fast CTA scans of the aorta. Of course, due to limitations of x-ray tube power, examinations of obese patients with the high-pitch techniques may be challenging. High-scan speed and correspondingly short-scan times are also helpful in pediatric radiology. ECG-triggered high-pitch scans have been used for comprehensive thorax examinations in the emergency room and in the planning and/or checking of TAVR procedures, because they provide adequate visualization of the coronary arteries, the aorta, and the iliac arteries in one scan at low radiation dose (see Fig. 16). The very short total scan time may allow for a reduction of the amount of contrast agent administered.

With DSCT dual-energy data can be acquired by simultaneously operating both x-ray tubes at different kV-settings, e.g., 80 and 140 kVp.^20^ Spectral separation can be improved by additional prefiltration of the high-kVp beam with the goal of better material quantification at reduced radiation dose by means of a tin filter that can be moved into the beam when needed and moved out for non-DE applications.^26^

Despite their clinical benefits, DSCT systems need to cope with a number of challenges. One major challenge for image reconstruction is data truncation: for a compact gantry design, one detector A covers the entire SFOV (Ø50  cm), whereas the other detector B is restricted to a smaller, central field of view (FOV) (see Fig. 13). Consequently, the projection data of detector B are truncated if the scanned object extends beyond the central FOV, and the data need to be extrapolated to avoid truncation artefacts in the images. Data acquired with detector A are used to extrapolate the truncated projections of detector B.

**Fig. 17:**
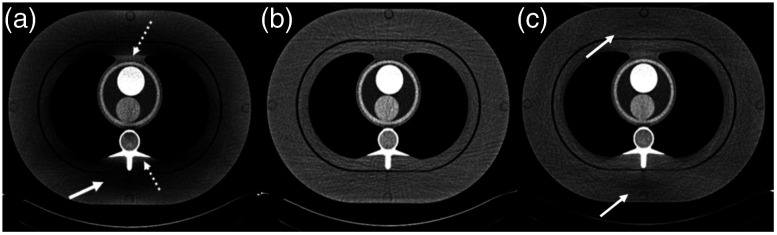
Images of an anthropomorphic thorax phantom with heart insert, scanned on a DSCT system. The x-ray beamwidth in the z direction was 38.4 mm at isocenter. FoV 420 mm, window width 300 HU, window center 40 HU. (a) No scatter correction. The arrows indicate scatter artifacts due to direct scatter and cross scatter. (b) Measurement-based scatter correction. (c) Model-based scatter correction. Images with modification from Ref. 27.

Another challenge is cross-scattered radiation, i.e., scattered radiation from x-ray tube B detected by detector A and vice versa. Cross-scattered radiation causes artefacts and degrades the contrast-to-noise ratio of the images. It can result in incorrect material decomposition and material classification in dual-energy scans. Cross scatter requires adequate correction.^27^ The most straightforward correction approach is to directly measure the cross-scattered radiation in detectors A and B and to subtract it from the measured signal. This technique requires additional detector elements on each detector outside the direct beam and is implemented in the second-generation DSCT. An alternative to direct measurement is model-based cross-scatter correction. The primary source of cross-scattered radiation is Compton scatter at the object surface, knowledge of the surface is therefore sufficient to predict cross scatter. The object surface, however, can be readily determined by analyzing the outline of the raw data sinogram. This technique is realized in the first-generation DSCT. Prestored cross-scatter tables for objects with similar surface shapes are used for an online correction of the cross-scattered radiation. The results of both measurement-based and model-based cross-scatter correction are shown in Fig. 17. In the third-generation DSCT, a correction based on a simplified Monte-Carlo simulation of cross scatter is implemented.

## 6 Dual-Energy Approaches

**Fig. 18:**
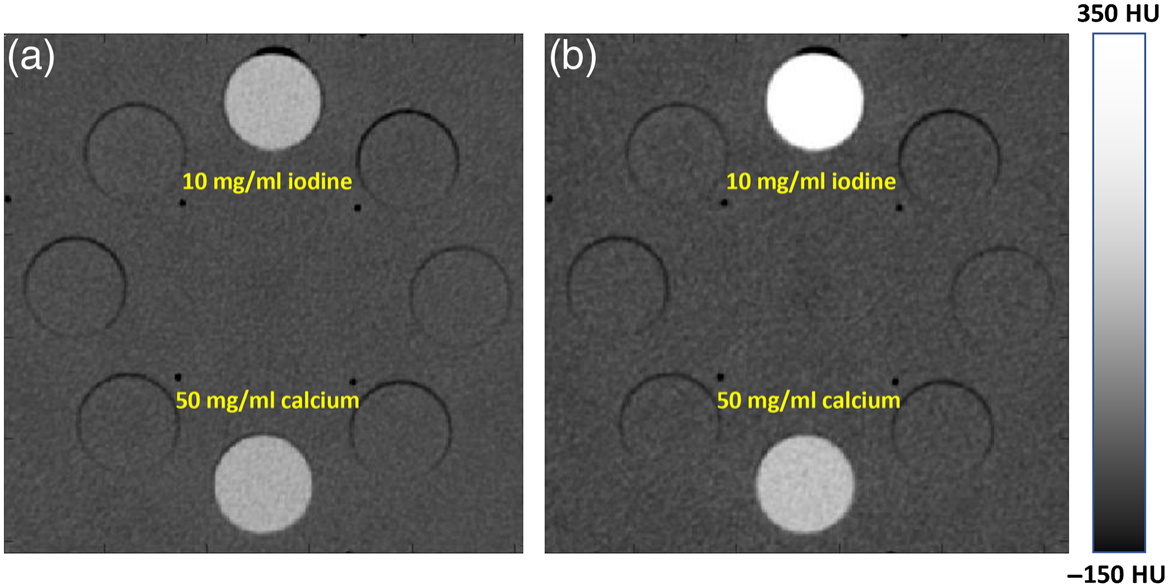
Illustration of material differentiation with DECT on a Gammex phantom (window width 500 HU, window center 100 HU): (a) 140 kVp image and (b) 80 kVp image.

In recent years, the use of dual-energy CT (DECT) in clinical practice has increased steadily. Although the concept of DECT was conceived not long after the invention of the CT itself, technical challenges associated with the CT hardware and software have prevented it from becoming a clinically useful tool.^28^^–^^30^ The advantage of DECT is its ability to provide information beyond the density of the scanned object and the potential to differentiate materials. For illustration, CT scans of a Gammex phantom with two different types of inserts (one with 10  mg/ml iodine and water mixture and the other with 50  mg/ml calcium and water mixture) were performed. When the phantom was scanned with 140 kVp setting, both insets present with identical CT numbers as shown in Fig. 18(a). Clearly, it is impossible to differentiate these insets solely based on this image. When the same phantom was scanned with 80 kVp, however, the CT numbers of the reconstructed inserts are significantly different, with the iodine insert showing a higher intensity as depicted in Fig. 18(b).

**Fig. 19:**
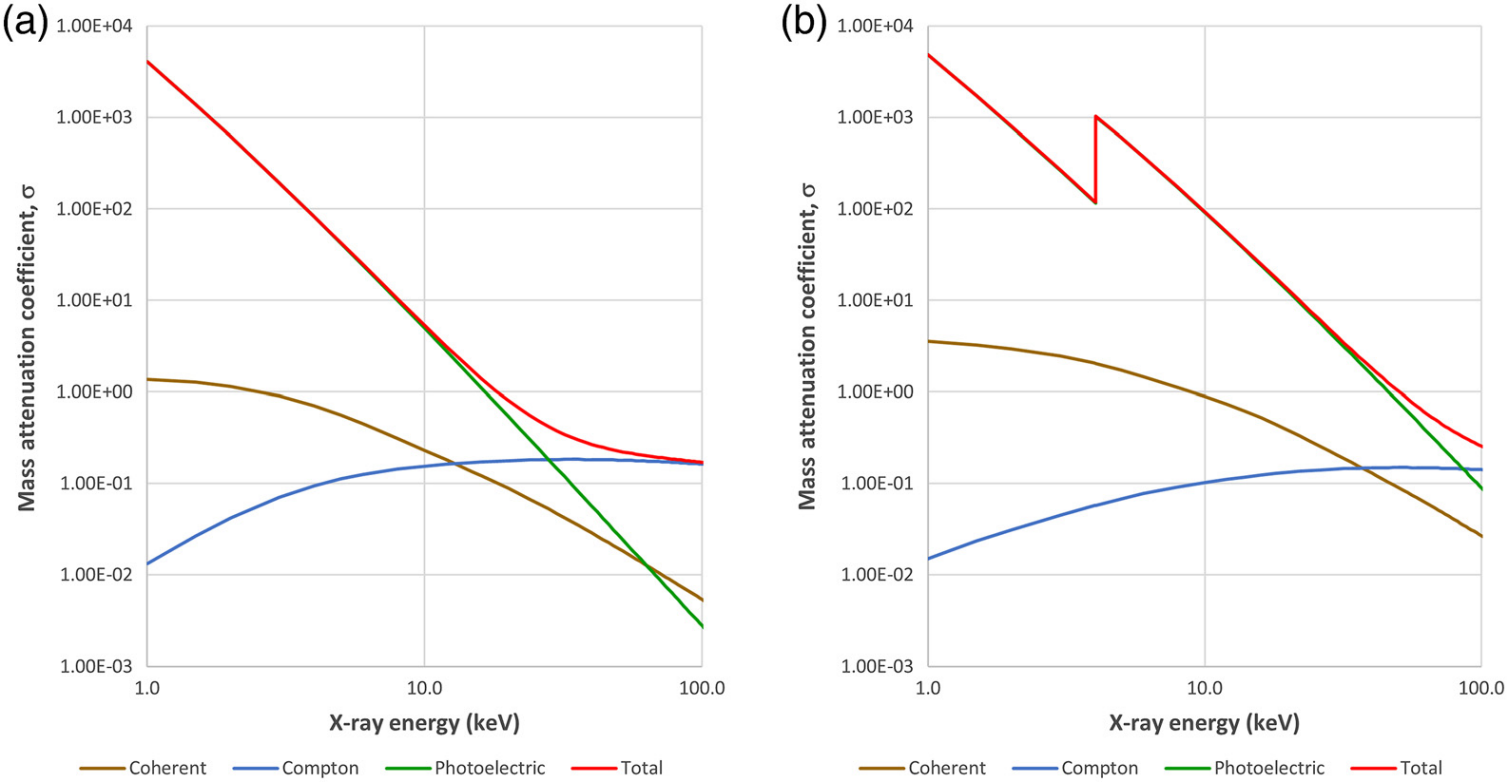
X-ray mass attenuation coefficients as a function of x-ray photon energy: (a) water and (b) calcium.

DECT takes advantage of the different x-ray photon interactions with matter, mainly the photoelectric effect and Compton scattering (coherent interactions can be ignored). In photoelectric interaction, the original x-ray photon ceases to exist while in Compton interaction a scattered x-ray photon is generated (For human body, x-ray fluorescence can be ignored). As illustrated by Fig. 19, photoelectric interactions (green curve) dominate for lower x-ray energy photons and Compton scattering (blue curve) becomes more significant for higher energies photons. In addition, the relative contributions of photoelectric and Compton varies with material. Therefore, to take advantage of these characteristics, DECT collects CT data at both low and high energies and the two unknowns (for photoelectric and Compton) can be solved for each voxel in the object.

**Fig. 20:**
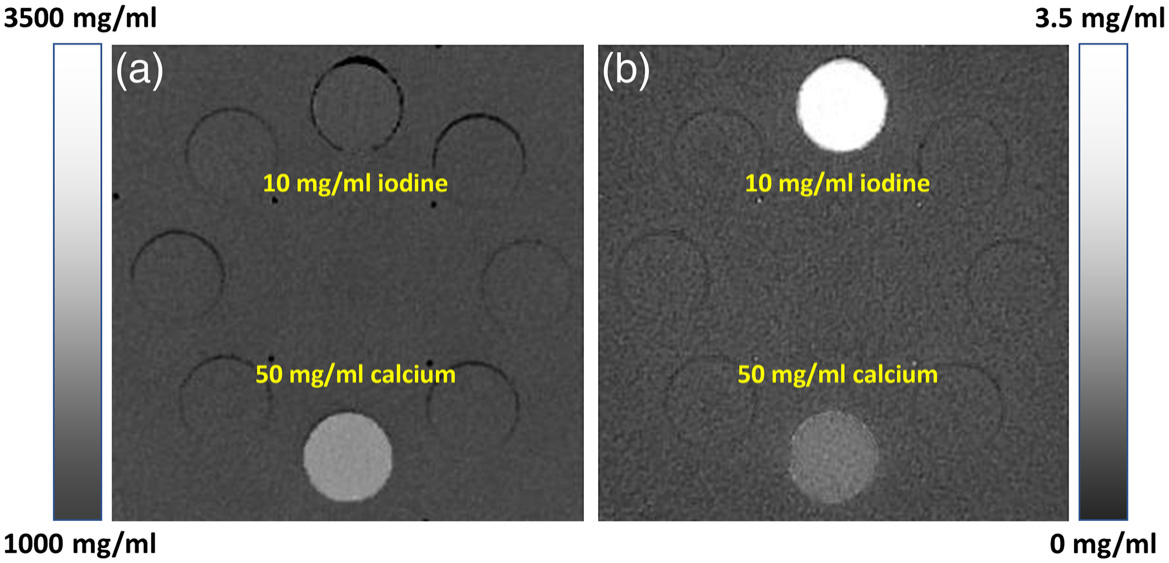
Illustration of material decomposition on a Gammex phantom: (a) water (iodine) image in mg/ml and (b) iodine (water) image in mg/ml.

Although it would be theoretically interesting to examine the photoelectric and Compton images, they are somewhat difficult to interpret in a clinical context. It can be shown, however, that the attenuation characteristics of any material can be characterized as the linear combination of two “basis” materials (e.g., water and iodine).^29^ This process is called the material decomposition where any material is “decomposed” into two other materials. Therefore, instead of solving for photoelectric and Compton images, one solves for density images of two basis materials with clear physical or clinical meaning. For illustration, Fig. 20 depicts the same Gammex phantom shown in Fig. 18 scanned and reconstructed with DECT using water and iodine as the basis material pair. In the water (iodine) image [Fig. 20(a)], the iodine component is removed [similarly, water component is removed in the iodine (water) image shown in Fig. 20(b)]. This leads to the density of the 10-mg/ml iodine insert being similar to the pure water background in Fig. 20(a). On the other hand, the 50-mg/ml calcium insert has contributions to both water (iodine) and iodine (water) images since calcium is neither water nor iodine. If we had selected water and calcium as the basis material pair, the water (calcium) image of the 50-mg/ml calcium insert would have similar density as the pure water background.

There are different ways of acquiring the dual-energy data and the acquisition can be classified as “source-driven” or “detector-driven.” In the source-driven approach (x-ray tube with additional filtration), the input x-ray spectrum is modified to provide different x-ray energy spectra to accomplish the dual-energy acquisition while the detector does not provide spectral information. For the detector-driven approach, on the other hand, the detector provides the needed information regarding the x-ray spectrum and the x-ray source does not change during data acquisition. Table 1 outlines some of the approaches used in the DECT data collection. There are pros and cons in each approach and given the limited scope of this paper, interested readers can refer to Ref. 31 for details.

**Table 1:** Outline of different approaches to dual-energy data acquisition.

Category	Acquisition	Description
Source-driven	Rotate–rotate	Low- and high-kVp data are collected sequentially either in step-and-shoot or helical mode
	Beam filtration	Different x-ray filtration is provided as part of prepatient collimation to produce different input x-ray spectra
	Dual source	Two sets of x-ray tube and detector are used in the data collection where one set produces low-kVp and one set produces high-kVp data
	Fast kVp switching	Low- and high-kVp are rapidly switched at the x-ray source such that interlaced project samples are collected
	Slow kVp switching	Low- and high-kVp are switched every n-views and an algorithmic approach is used to estimate the missing data
Detector-driven	Dual layer	Detector consist of two distinct layers with the top layer detects primarily low-energy x-ray photons and the bottom layer detects primarily high-energy photons

For DECT, two important performance parameters are the accuracy and precision of the reconstructed density images.^32^ For example, the ability to quantify the concentration of iodine is an important performance measure for DECT. Quantitation can be impacted by many factors, such as energy separation between high- and low-energy data, noise in the collected data, and the reconstruction algorithm.

**Fig. 21:**
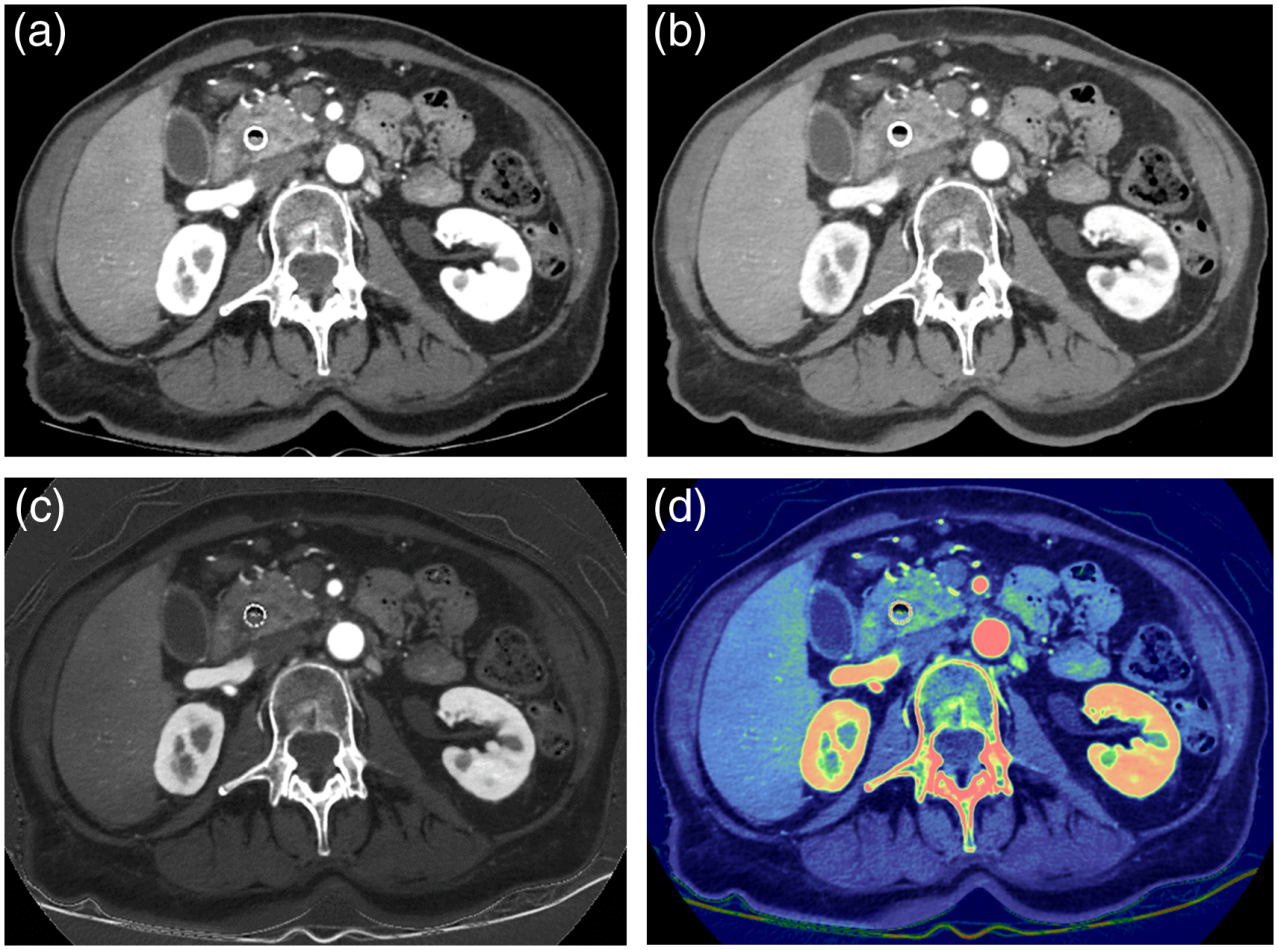
Illustration of DECT to improve the contrast-to-noise ratio and the lesion detectability for a patient with pancreatic cancer: (a) 40-keV virtual monochromatic image, (b) 70-keV virtual monochromatic image, (c) iodine (water) image, and (d) color-overlay image. Image courtesy of Dr. Nakul Gupta, Houston Methodist Hospital, USA.

DECT has wide clinical utility; it adds additional dimension to the CT image. Many researchers use a black-and-white TV set versus color TV set analogy to characterize the difference between a single-energy CT and DECT since DECT adds spectral or “color” information. The added information can provide distinct clinical benefits. For example, one of the clinical applications of DECT is to enhance the contrast of iodinated vessels and pathologies. Based on the characteristics of x-ray interaction with matter, most material exhibits higher attenuation to lower energy x-rays (note the rapid increase in mass attenuation coefficient for water as the x-ray energy decreases in Fig. 19). Therefore, by synthesizing CT scans collected with a lower energy monochromatic x-ray source, the contrast of the object can be significantly enhanced. This is illustrated in Figs. 21(a) and 21(b). The contrast-to-noise improvement of 40 over 70 keV is apparent. In addition, since iodine can be “isolated” in the iodine (water) image, it can be used to highlight or enhance the appearance of iodine contrast uptake in the body to improve the visibility of certain pathologies. Figure 21(d) depicts a color overlay of the iodine (water) density image over the 70-keV image. Compared to the monochromatic images, the lesion is better visualized in the color-overlayed image and such visualization can help radiologists to focus on particular areas and provide improved confidence in diagnosis.

**Fig. 22:**
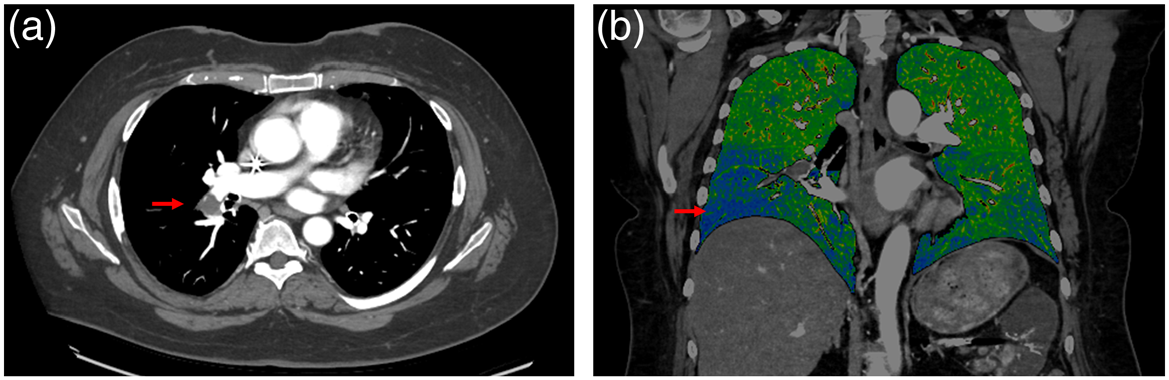
Illustration of DECT for pulmonary emboli detection (a) 70-keV image to show thrombus (b) color-overlayed image to highlight the affected lung region. Image courtesy of Dr. W. Dennis Foley, Froedtert & Medical College of Wisconsin, USA.

Another example of DECT application is helping radiologists to easily identify the area of perfusion defect in the case of pulmonary embolism. Thrombus often leads to deficiencies in the blood perfusion in the affected area of the lung. Since DECT can provide a map of the iodine, lack of iodine uptake in the lung can be readily identified. Figure 22 depicts a patient study with positive pulmonary emboli where the region highlighted in blue identifies the affected area. Such areas can be traced nicely to the detected thrombus shown by the red arrow in Fig. 22(a).

## 7 Photon Counting CT

Photon-counting detectors are a new technology with the potential to provide CT data at very high spatial resolution, without electronic noise and with inherent spectral information. Photon counting detectors were evaluated in prototype CT benchtop systems more than 10 years ago.^33^ The performance of the detectors used in these early systems, however, was not adequate for clinical CT imaging, mainly because they did not tolerate the high x-ray flux rates of medical CT. Significant recent progress in detector material synthesis has meanwhile enabled the installation of preclinical whole-body photon-counting CT prototypes for human use. Interested readers may refer to reviews as found in Refs. 34–38.

**Fig. 23:**
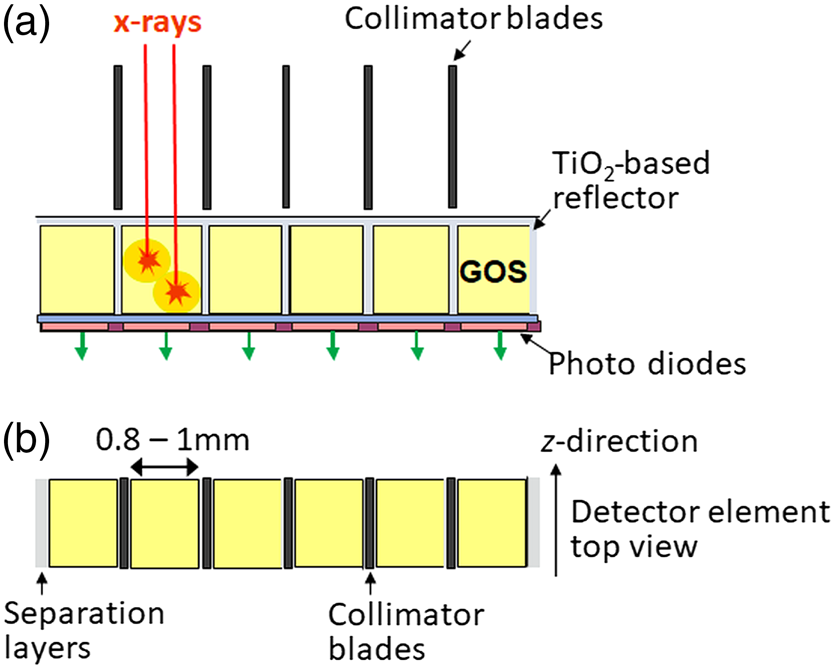
Schematic drawing of an energy-integrating scintillator detector: (a) side view and (b) top view. The z direction is the patient’s longitudinal direction. Detector cells made of a scintillator such as GOS absorb the x-rays (red arrows) and convert their energy into visible light (orange circles).

To understand the benefits of photon-counting CT detectors, it is helpful to briefly review the properties of solid-state scintillation detectors which are used in all current medical CT scanners. They consist of individual detector cells with a side length of 0.8 to 1 mm, made of a scintillator (e.g., gadolinium-oxide or gadolinium-oxysulfide [GOS], Lumex, and LuTag^39^) with a photodiode attached to its backside (see Fig. 23). The absorbed x-rays produce visible light in the scintillator which is detected by the photodiode and converted into an electrical current. The intensity of the scintillation light and, as a consequence, the amplitude of the induced current pulse is proportional to the energy E of the absorbed x-ray photons. All current pulses registered during the measurement time of one projection are integrated. Lower energy photons, which carry most of the soft-tissue low-contrast-information, contribute less to the integrated detector signal than higher energy photons. This energy-weighting reduces the contrast-to-noise ratio mainly in contrast enhanced CT scans because the x-ray absorption of iodine is highest at lower energies (above its K-edge at 33 keV).

The low-level analog electric signal of the photodiodes is corrupted by electronic noise which becomes larger than the quantum noise (Poisson noise) at low x-ray flux and causes a disproportional increase of image noise and instability of CT-numbers (e.g., in low-dose lung CT imaging). This strong noise increase and the drift of CT-numbers set a limit to potential further radiation dose reduction in medical CT.

The individual detector cells are separated by optically opaque reflection layers based on ${\mathrm{TiO}}_{2}$ or ${\mathrm{Cr}}_{2}O_{3}$ to prevent optical crosstalk (see Fig. 23). They have a width of about 0.1 mm and reduce the geometric dose efficiency of the detector. X-ray photons absorbed in the separation layers do not contribute to the measured signal even though they have passed through the patient. Current medical CT detectors with an active cell size of about $0.8\times 0.8\;{\mathrm{mm}}^{2}$ to $1\times 1\;{\mathrm{mm}}^{2}$ have a geometric dose efficiency of 70% to 80%. If the width of the separation layers is kept constant, significantly reducing the size of the cells (in order to increase the spatial resolution) would further decrease the geometric efficiency—therefore, it is challenging to increase the spatial resolution of solid-state scintillation detectors.

**Fig. 24:**
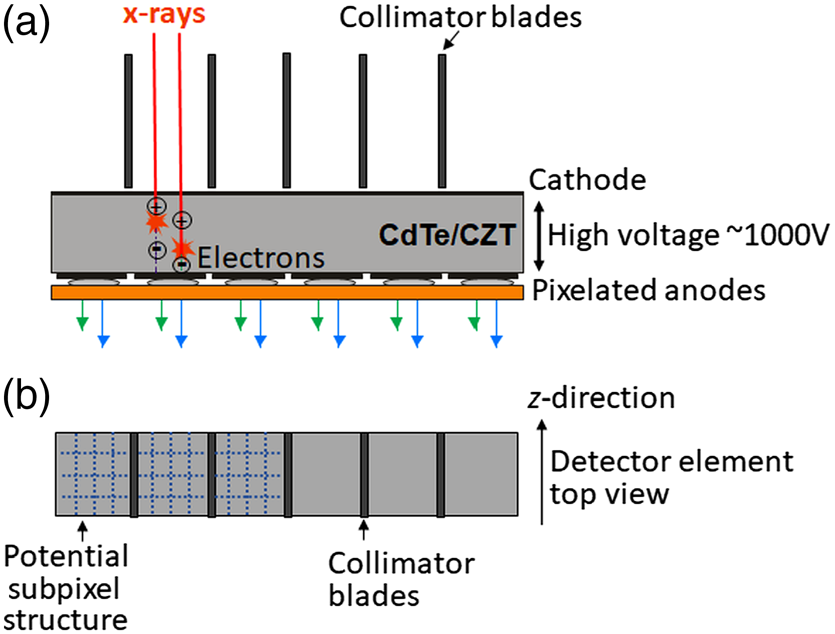
Schematic drawing of a direct converting photon-counting detector: (a) side view and (b) top view. The x-rays (red arrows) absorbed in a semiconductor such as CdTe or CZT produce electron–hole pairs that are separated in a strong electric field between cathode and pixelated anodes. A potential subpixel structure is indicated for the three left detector cells. The pixelated anodes must then be correspondingly structured (not shown here in order not to overload the drawing).

**Fig. 25:**
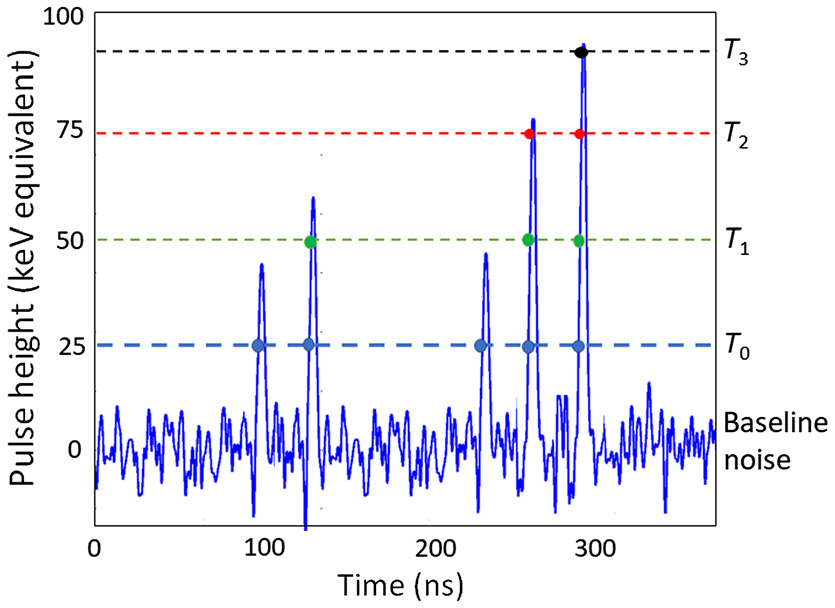
The signal pulses induced by absorbed x-rays in a photon-counting detector are counted as soon as they exceed a threshold $T_{0}$ (dashed blue line, “counting” is indicated by a blue dot). $T_{0}$ has a typical energy of 25 keV, well above the low-amplitude baseline noise. Three additional thresholds at higher energies ($T_{1}$ at 50 keV, $T_{2}$ at 75 keV, and $T_{3}$ at 90 keV) are also indicated—simultaneous read-out of the counts at various energy thresholds (in this example 4) provides spectrally resolved detector signals.

Photon-counting detectors are made of semiconductors such as cadmium-telluride (CdTe), cadmium-zinc-telluride (CZT), or silicon (Si). High voltage (800 to 1000 V) is applied between the cathode on top and pixelated anode electrodes at the bottom of the semiconductor layer (see Fig. 24). The absorbed x-rays produce electron–hole pairs which are separated by the strong electric field. The electrons drift to the anodes and induce short-current pulses in the order of nanoseconds (ns). A pulse-shaping circuit transforms them to voltage pulses with an FWHM of 10 to 15 ns. The pulse-height of the voltage pulses is proportional to the energy E of the x-ray photons. As soon as these pulses exceed a threshold, they are counted (see Fig. 25).

**Fig. 26:**
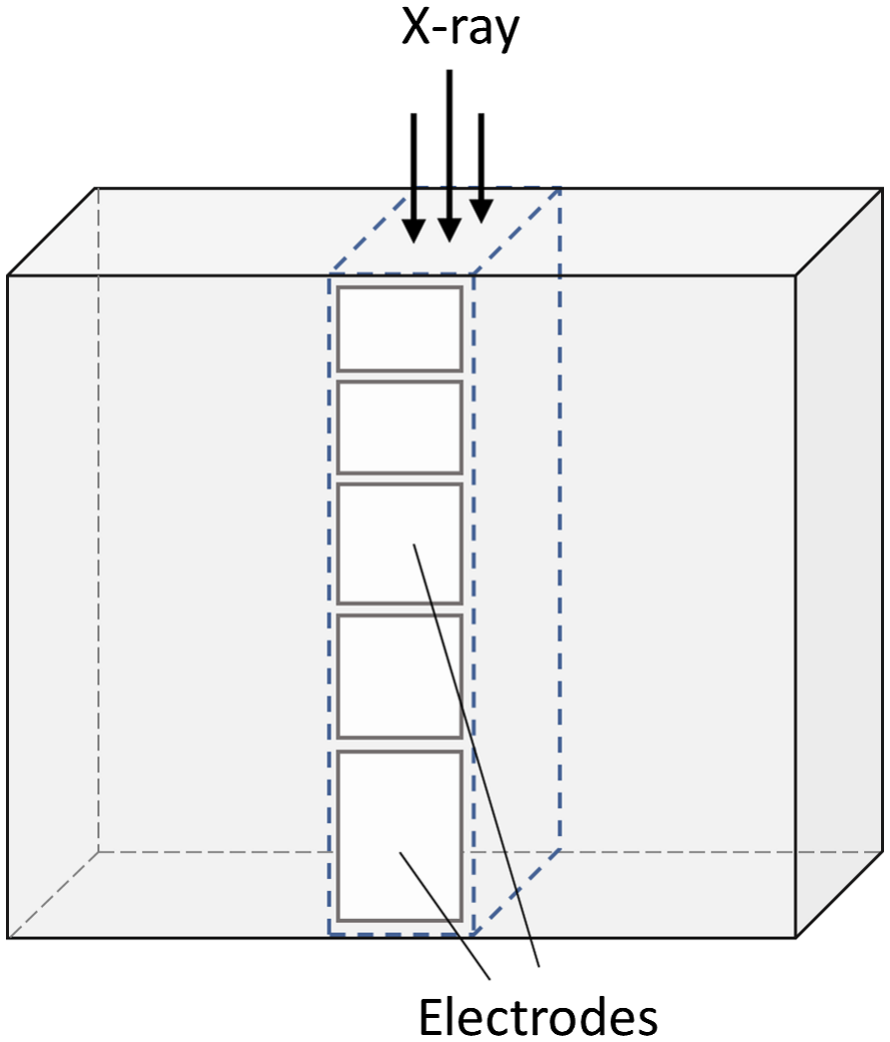
Edge-on realization of a photon counting detector. This design is suitable for detector materials such as silicon, which have lower x-ray attenuation coefficients. The distribution of interactions over a larger volume potentially helps to mitigate pulse pileup effects.

Depending on the material, the detector consists of a 1.4- to 30-mm thick semiconductor layer. Thinner layers are sufficient for CdTe- and CZT-based CT detectors because of their high atomic number. Silicon-based CT detectors, such as that shown in Fig. 26, use thicker layers owing to the lower atomic number of the detector material. The larger thickness enables photon counting to be partitioned into a larger volume to potentially mitigate the pulse pileup effect.^40^

Photon-counting detectors have several advantages compared to solid-state scintillation detectors. The detector cells are defined by the strong electric field between common cathode and pixelated anodes (Fig. 24), there are no additional separation layers. The geometrical dose efficiency is only reduced by the unavoidable antiscatter collimator blades or grids. Different from scintillator-based detectors each “macropixel” confined by collimator blades can be divided into smaller subpixels, which are read-out separately to increase spatial resolution [see Fig. 24(b)].

All current pulses induced by absorbed x-rays are counted during the measurement time of one projection as soon as they exceed a threshold energy. Low-amplitude baseline noise is well below this level and does not trigger counts—even at low-x-ray flux only the statistical Poisson noise of the x-ray quanta is present in the signal. CT scans at very low-radiation dose or CT scans of obese patients show therefore less image noise, less streak artifacts, and more stable CT-numbers than the corresponding scans with scintillation detectors. Radiation dose reduction beyond today’s limits seems possible.

There is no down-weighting of lower energy x-ray photons as in solid-state scintillation detectors. Photon-counting detectors can therefore provide CT images with potentially improved CNR, in particular in CT scans with iodinated contrast agent.

**Fig. 27:**
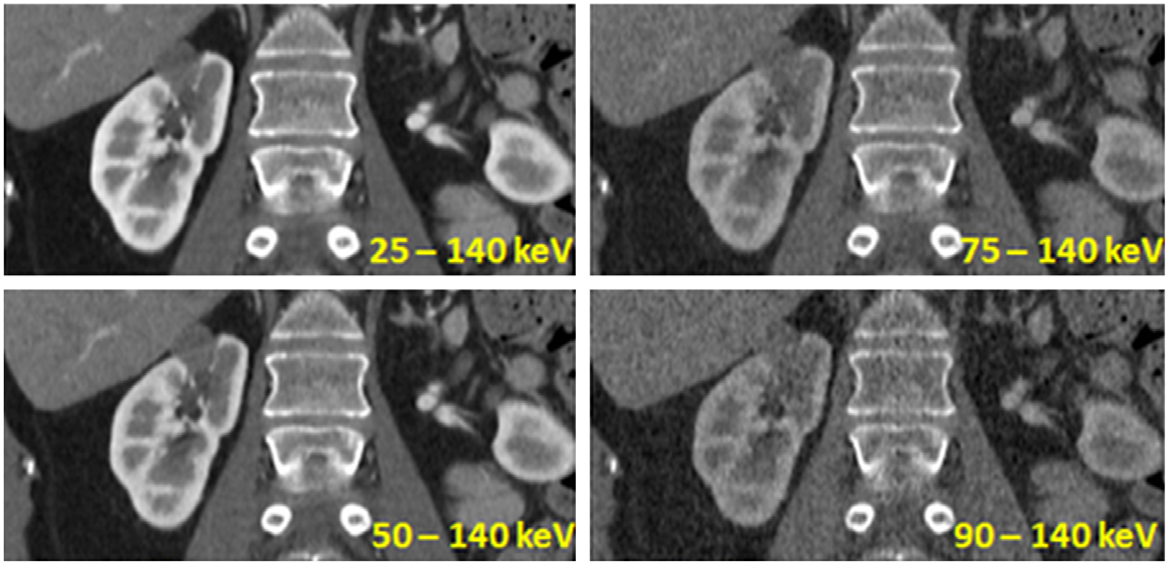
Contrast-enhanced kidney scan acquired with a preclinical photon counting CT prototype with four low-energy thresholds (25, 50, 75, and 90 keV) as indicated in Fig. 25, operated at an x-ray tube voltage of 140 kVp. The higher the lower energy threshold is, the lower is the iodine contrast, and the higher is the image noise in the reconstructed images, because fewer low-energy x-ray photons contribute to the image. Images courtesy of National Institutes of Health (NIH), Bethesda, MD, USA.

In a more advanced readout mode, several counters operating at different threshold energies are introduced for energy discrimination (see Fig. 25). In this example, four different energy thresholds $T_{0}$, $T_{1}$, $T_{2}$, and $T_{3}$ are used, and the photon-counting detector simultaneously provides four signals $S_{0}$, $S_{1}$, $S_{2}$, and $S_{3}$ with different lower energy thresholds. CT images reconstructed from these raw data are shown in Fig. 27. By subtracting the detector signals with adjacent energy thresholds, “energy bin” data can be produced. Energy bin $b_{0}=S_{0}-S_{1}$, as an example, contains all x-ray events detected in the energy range between $T_{0}$ and $T_{1}$.

Silicon detectors have been realized with eight energy bins, which may allow additional flexibility in terms of energy-weighting of signals to optimize specific task-based performance, as well as accommodating a larger number of possible simultaneously administered contrast materials.^41^^,^^42^

Similar to other detector-driven approaches for dual energy data acquisition, CT systems with photon counting detector enable spectrally resolved measurements and material differentiation in any CT scan by the simultaneous read-out of CT data in different energy bins. Today’s established dual-energy applications—mainly based on decomposition into two base materials such as iodine and water—are routinely feasible. Data acquisition with more than two energy bins enables multi-material decomposition if a material with K-edge in the relevant energy range of CT (40 to 100 keV), such as gadolinium, is added to the two base materials. Unfortunately, three- or more-material decomposition with CT data in three or more energy bins will be limited to clinical scenarios, in which K-edge elements have been administered to the patient to separate two contrast agents (e.g., iodine and gadolinium or iodine and bismuth) or other heavy elements (e.g., tungsten or gold nanoparticles).

**Fig. 28:**
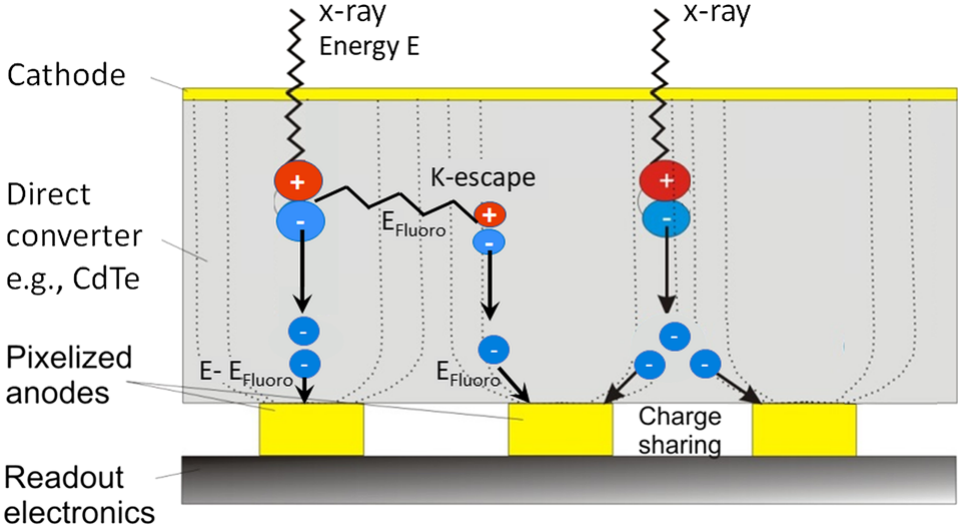
Schematic illustration of charge sharing at pixel boundaries and energy loss due K-escape, which lead to double counting of x-ray pulses at wrong energies and reduction of spectral separation. $E_{\text{fluoro}}$ is the K-shell fluorescence x-ray energy.

Compared to established dual-energy acquisition techniques, photon-counting detectors are often assumed to provide better energy separation and less spectral overlap. However, unavoidable physical effects reduce the energy separation. The current pulses induced by x-rays absorbed close to pixel borders are split between adjacent detectors cells (“charge sharing”). This leads to erroneous counting of one high-energy x-ray photon as several lower-energy hits. Cd and Te have K-edges at 26.7 and 31.8 keV, respectively. Incident x-rays likely ionize K-electrons of the detector material. The empty K-shells are immediately refilled, and characteristic x-rays at the K-shell fluorescence energy $E_{\text{fluoro}}$ are released which are reabsorbed and counted in the detector cell itself or in neighboring cells (“K-escape”). The incident x-rays are counted at lower energy $E-E_{\text{fluoro}}$. (A resulting peak in the detector signal is called “K-escape peak.”) In summary, high-energy x-ray photons may be wrongly counted at lower energies, and spectral separation as well as spatial resolution may be reduced. Charge sharing, fluorescence, and K-escape are illustrated in Fig. 28. For a realistic detector model including charge sharing, fluorescence, and K-escape, the spectral separation with two energy bins is probably equivalent to that of a dual-kVp scan with optimized prefiltration.

Medical CTs are operated at high x-ray flux rates up to $10^{9}$ counts per second per ${\mathrm{mm}}^{2}$—if the detector pixels are too large, too many x-ray photons hit a pixel too closely in time to be registered separately. Several overlapping pulses are then counted as one hit but at a higher energy (“pulse pile-up”). Pulse pile-up leads to non-linear detector count rates and finally to detector saturation. Even though the signal can be linearized before the onset of saturation, significant quantum losses, increased image noise, and reduced energy discrimination cannot be avoided. A way out of this is a reduction of the size of the detector cells—however, smaller cells lead to more charge sharing and K-escape. Finding the optimum size of the detector cells to balance pulse pile-up, charge sharing, and K-escape is a challenging task in designing a photon-counting detector. Edge-on silicon detectors shown in Fig. 26 are less impacted by K-escape because the K-edge of silicon is well below the diagnostic energy range. However, they are affected by Compton scatter within the detector, which produces lower energy scattered photons and increases crosstalk. These scatter events, which are also used in the image formation process, are generally well-separated from primary events in terms of energy and may be discriminated by the detector.

Photon-counting detectors are a promising new technology for future medical CT. Currently, prototypes are used to evaluate the potential and limitations of photon-counting CT in clinical practice.

Silicon-based photon counting detectors were first evaluated for dedicated breast CT imaging, but the scope was soon extended to other applications. Meanwhile, a prototype single-source CT scanner with a full-field-of-view silicon-based photon-counting detector capable of patient scanning has been presented.^41^

There are several experimental prototype CT-systems equipped with CdTe- or CZT-detectors. A small-bore spectral micro-CT based on a Medipix-detector with eight energy channels has been translated to a large-bore photon counting CT,^43^ however, no further results have been published. Recently, a single source spectral photon counting CT system with CZT-detector and five programmable energy thresholds has been installed.^44^ The scanner acquires data from 64 rows to reconstruct a 50-cm FOV with a z coverage of 17.6 mm at the isocenter and is based on a previous system with smaller FOV and z coverage.^45^ This system was evaluated both with phantoms and with animal scans, demonstrating improved spatial resolution^45^ and spectral capabilities, such as differentiation of several contrast agents.^46^ A hybrid dual-source CT scanner prototype equipped with a conventional scintillation detector and a CdTe photon counting detector was described and first evaluated in Ref. 47. Recently, another single-source CdTe photon counting CT scanner with four energy thresholds, an FOV of 50 cm at the isocenter and a z coverage up to 57.6 mm (either 120×0.2  mm or 144×0.4  mm collimation) has been installed in three preclinical settings.^38^ The system is capable of patient scanning using typical clinical scan protocols.^48^

**Fig. 29:**
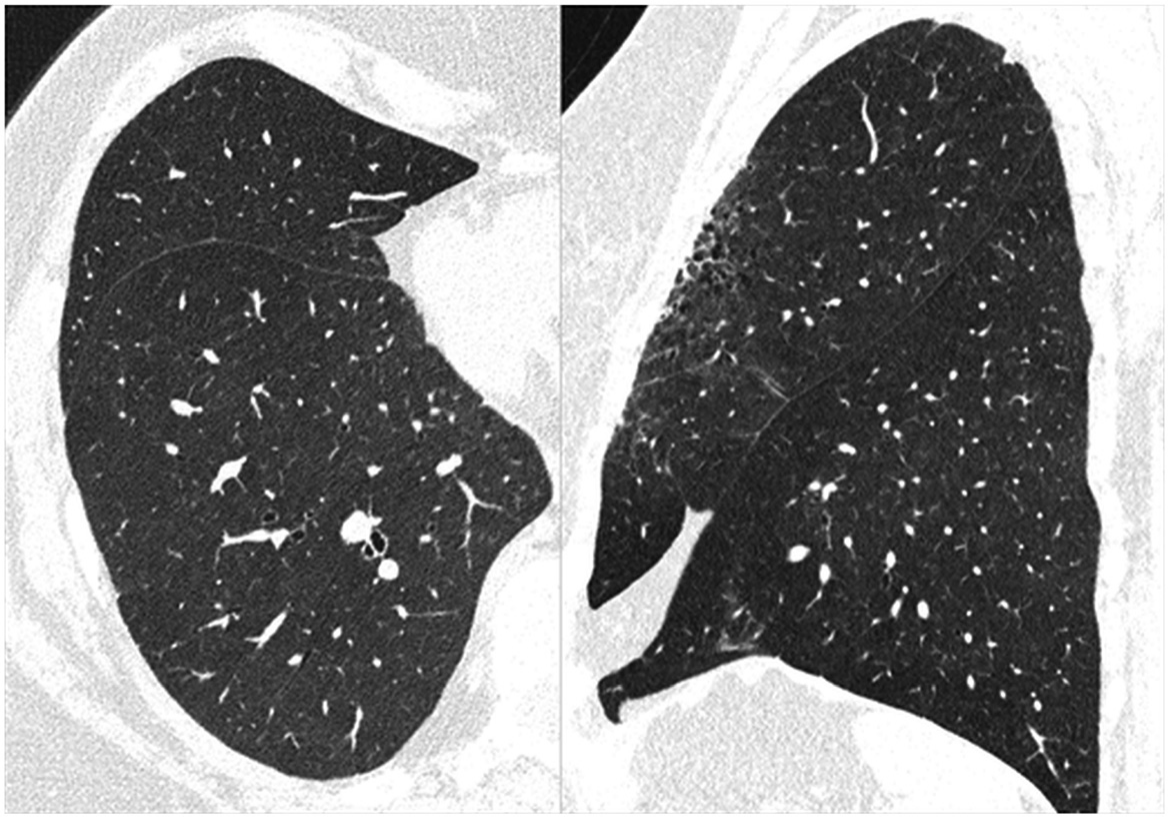
Lung images of a 74-year-old woman with breast cancer and signs of fibrosis after radiation therapy, acquired with a single-source CT prototype with photon-counting detector. Data acquisition: 120×0.2  mm collimation, 0.3 s rotation time, ${\mathrm{CTDI}}_{\mathrm{vol}}=3.89\;\mathrm{mGy}$, DLP=126  mGycm. Image reconstruction: sharp convolution kernel, 1024×1024 image matrix, 0.4 mm slice width. Excellent visualization of fibrosis and fine details such as fissures is achieved. Images courtesy of Dr. J. Ferda, Pilsen University, Czech Republic.

**Fig. 30:**
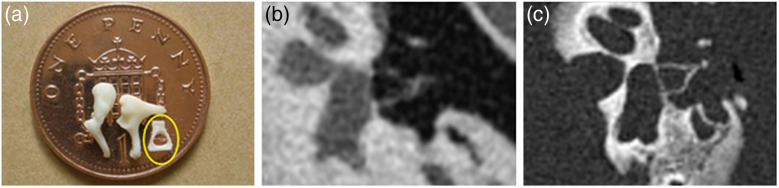
(a) Bones of the middle ear—the stapes (yellow circle) has a size of about 2  mm×3  mm. Specimen image acquired with (b) a state-of-the-art medical CT and (c) a single-source CT prototype with photon-counting detector. Data acquisition: 120×0.2  mm collimation. Spatial resolution is significantly improved. Images courtesy of Dr. A. Persson, Center for Medical Image Science and Visualization (CMIV), Linköping, Sweden.

**Fig. 31:**
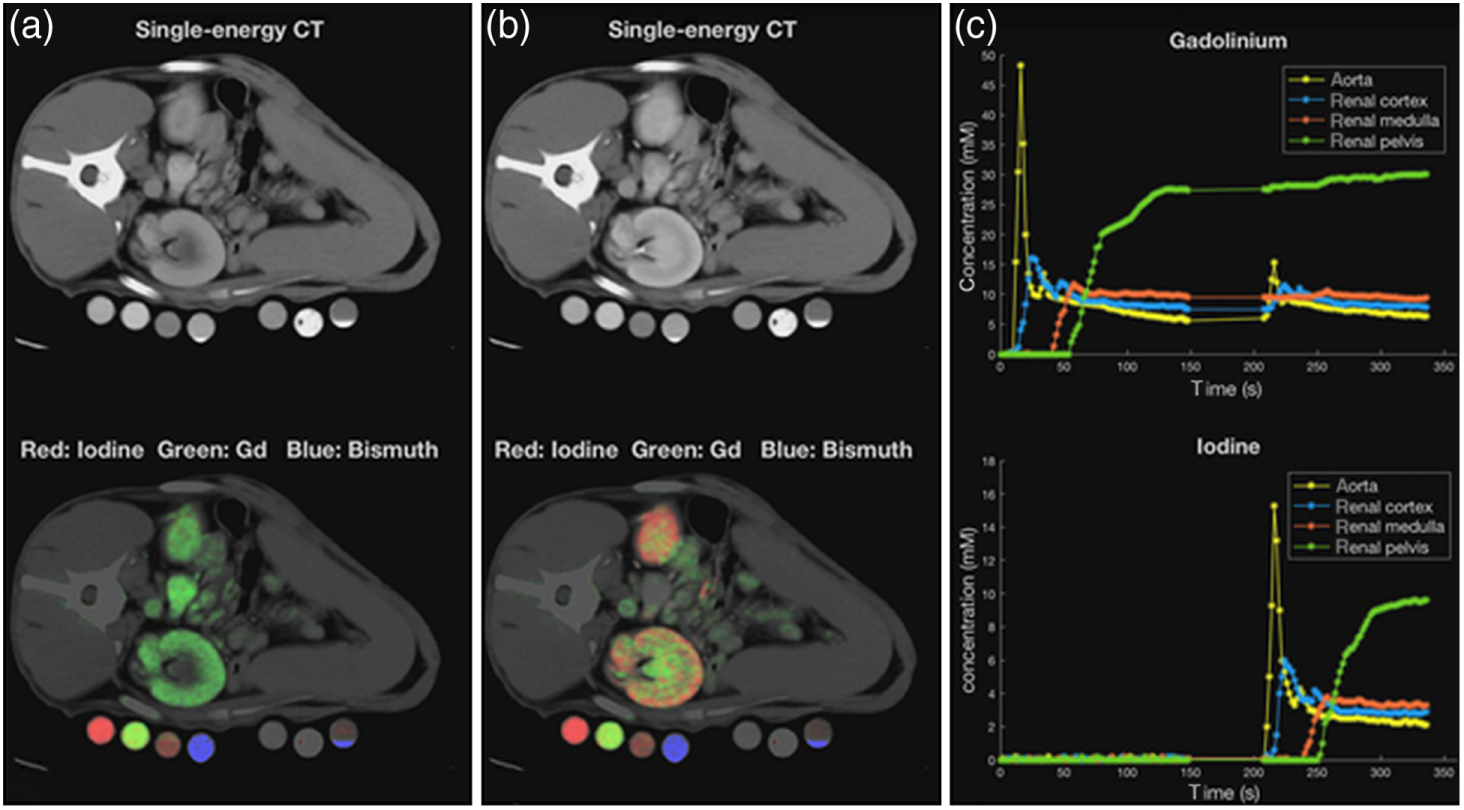
Simultaneous imaging of three different contrast agents (iodine, gadolinium, and bismuth) by multi-material decomposition in a dog model. Scan data were acquired with the preclinical photon counting CT prototype and read-out in four energy bins (25 to 50, 50 to 75, 75 to 90, and 90 to 140 keV). Bismuth was administered more than one day prior to scanning. Intravenous administration of gadolinium-based contrast agent was followed by intravenous administration of iodine-based contrast agent after 3 min to simultaneously visualize different phases of renal enhancement. (a) Image acquired at 30 s after start of gadolinium injection at the peak of gadolinium enhancement in the renal cortex. (b) Image acquired at 220 s at the peak of iodine enhancement in the renal cortex. (c) Enhancement curves of gadolinium and iodine in the aorta, renal cortex, medulla, and pelvis. Courtesy of R. Symons, NIH, Bethesda, MD, USA (see also Ref. 49).

A key benefit of photon-counting CT is improved spatial resolution. The achievable image quality with a photon-counting detector in high-resolution chest CT is demonstrated in Fig. 29. Figure 30 illustrates the resolution improvement for temporal bone anatomy. Another key benefit is the routine availability of spectral CT data in more than two energy bins, which enables multi-material imaging under certain conditions. The feasibility of simultaneous material decomposition of three contrast agents (bismuth, iodine, and gadolinium) *in vivo* in a canine model by a photon counting CT with four energy bins is demonstrated in Fig. 31.^49^ Once remaining challenges of photon-counting CT have been mastered, this technology has the potential to bring clinical CT to a new level of performance.
